# Simulation-Based Study on the Performance of NSM-CFRP Strengthening in Prestressed Concrete T-Beams Under Seismic Loading

**DOI:** 10.3390/ma18184386

**Published:** 2025-09-19

**Authors:** Yanuar Haryanto, Hsuan-Teh Hu, Anggun Tri Atmajayanti, Fu-Pei Hsiao, Laurencius Nugroho, Nanang Gunawan Wariyatno

**Affiliations:** 1 Department of Civil Engineering, Universitas Jenderal Soedirman, Purwokerto 53122, Indonesia; nanang.wariyatno@unsoed.ac.id; 2 National Center for Research on Earthquake Engineering, Taipei 106, Taiwan; fphsiao@ncree.narl.org.tw; 3 Department of Civil Engineering, National Cheng Kung University, Tainan 701, Taiwan; laurenciusnugroho@gmail.com; 4 Department of Civil Engineering, Universitas Atma Jaya Yogyakarta, Sleman 55281, Indonesia

**Keywords:** CFRP, seismic loading, simulation study, NSM, prestressed concrete T-beams, retrofitting, structural resilience

## Abstract

Prestressed concrete structures are facing serviceability challenges due to rising live loads, material degradation, and seismic demands. Retrofitting with carbon fiber-reinforced polymer (CFRP) offers a cost-effective alternative to full replacement. This study presents a finite element (FE) modeling framework to simulate the seismic performance of prestressed concrete T-beams retrofitted in the negative moment region using near-surface-mounted (NSM) CFRP rods and sheets. The model incorporates nonlinear material behavior and cohesive interaction at the CFRP–concrete interface and is validated against experimental benchmarks, with ultimate load prediction errors of 4.41% for RC T-beams, 0.49% for prestressed I-beams, and 1.30% for prestressed slabs. A parametric investigation was conducted to examine the influence of CFRP embedment depth and initial prestressing level under three seismic conditions. The results showed that fully embedded CFRP rods consistently improved the beams’ ultimate load capacity, with gains of up to 10.84%, 16.84%, and 14.91% under cyclic loading, near-fault ground motion, and far-field ground motion, respectively. Half-embedded CFRP rods also prove effective and offer comparable improvements where full-depth installation is impractical. The cyclic load–displacement histories, the time–load histories under near-fault and far-field excitations, stiffness degradation, and damage contour analysis further confirm that the synergy between full-depth CFRP retrofitting and optimized prestressing enhances structural resilience and energy dissipation under seismic excitation.

## 1. Introduction

The fundamental principle of concrete prestressing is to counteract tensile stresses by applying a compressive force in advance, typically achieved using tensioned steel tendons in the form of strands, wires, or cables. This approach enhances the structural efficiency of concrete, which is inherently strong in compression but weak in tension [1,2,3]. Compared to conventional reinforced concrete (RC), prestressed systems offer improved control over service-level cracking and deflection, reduced structural weight, and more efficient use of high-strength steel [2,4]. These benefits have led to the widespread use of prestressing techniques in various engineering applications, and particularly in long-span bridges, precast elements, and marine structures [5,6,7,8,9,10]. Prestressed T-beams, which are often used in simply supported configurations, are common in medium-span systems [11,12]. However, as many such systems age, they are increasingly subjected to service conditions that exceed their original design parameters, including rising live loads and environmental degradation. In these cases, structural strengthening offers a more cost-effective and less disruptive alternative to full replacement, as it can restore strength and stiffness while extending service life [13,14].

Historically, strengthening techniques have relied heavily on steel-based materials due to their accessibility, affordability, and familiarity within engineering practice [11,15,16,17]. Steel plates, in particular, have been widely applied to prestressed beams by bonding them externally with high-performance adhesives, thus effectively serving as supplemental reinforcement. This method has been shown to improve load-carrying capacity, stiffness, and midspan deflection [18]. U-shaped configurations of steel plates have proven especially effective in controlling shear crack propagation. Despite these benefits, however, the long-term durability of steel-strengthened systems remains questionable in corrosion-prone environments. This issue is particularly acute in aging bridge infrastructure or chloride-exposed settings, where corrosion can progressively reduce the cross-sectional area of tendons, leading to a decline in prestress force and flexural rigidity. In severe cases, this may cause a shift from ductile modes of failure to brittle ones [13,19,20,21,22].

In the face of these durability concerns, fiber-reinforced polymer (FRP) composites have emerged as a preferred alternative to metallic materials. Since it offers excellent corrosion resistance, high tensile strength, and ease of application, FRP has gained popularity for use in both new construction and retrofitting scenarios [23,24]. The versatility of this material lies in its availability in multiple configurations, such as plates, sheets, rods, and ropes, allowing wide adaptability in both structural design and practical application [25,26,27]. Among the established techniques for FRP-based strengthening, the externally bonded (EB) system and the near-surface-mounted (NSM) approach are the two most widely adopted [28,29,30,31,32]. The EB technique strengthens structural elements by attaching FRP sheets or plates to their surfaces, which has been shown to markedly enhance flexural and shear performance, particularly with the use of U-wraps for web reinforcement [26,27]. However, performance at higher stress levels can be limited by premature debonding [33,34]. In contrast, in the NSM approach, FRP rods or strips are embedded into pre-cut grooves in the concrete surface, thus improving anchorage and mitigating the risk of debonding [35]. Numerous studies have confirmed that NSM systems generally outperform EB configurations in terms of load capacity and crack control.

Design standards, such as ACI 440 [36], have been established to guide the practical use of FRP systems, specifying requirements like the minimum groove depth for NSM applications. Specifically, a minimum groove depth equal to 1.5 times the diameter of the FRP rod is required to achieve full adhesive bonding, promoting effective stress transmission and strain compatibility. These conditions are essential for controlling bond behavior and governing failure modes [37,38,39,40]. Nevertheless, it is not always feasible to achieve the recommended embedment depth, due to structural or construction constraints. To address this issue, recent research has examined half-embedded strategies, particularly for beams in negative moment regions where tensile demands are high. Haryanto et al. [41,42] showed that RC T-beams retrofitted with half-embedded carbon-FRP (CFRP) rods exhibited a 24% increase in capacity compared to 38% for beams with full embedment, as the latter benefitted from greater bonded surface area. While both configurations had a similar initial stiffness, the half-embedded system was limited by early debonding. Han et al. [43] extended this work under non-reversed cyclic loading, and simulated fatigue conditions in continuous bridge spans. Their findings confirmed that although half-embedded systems provided substantial strength gains and energy dissipation, fully embedded rods ensured better cyclic durability. These conclusions were further supported by the work of Nugroho et al. [44], which observed that even half-embedded systems exhibited substantial strength gains and reduced cracking under high-rate loading. Collectively, these studies demonstrate that half-embedded systems, despite not being code-compliant, can offer a practical and structurally viable solution when full embedment is infeasible due to detailing limitations or field conditions.

In parallel to experimental efforts, finite element (FE) modeling has become a powerful approach for analyzing the nonlinear behavior of prestressed and strengthened concrete members. The FE method (FEM) enables high-resolution assessment of internal stress and strain distributions, particularly in areas where the use of direct instrumentation would be impractical or invasive. Pham and Hong [45] demonstrated the effectiveness of nonlinear FE modeling by accurately replicating load–deflection responses and strain development in prestressed RC beams. Similarly, Chira et al. [46] employed FEM to study wide prestressed concrete beams, offering insights into the force distribution, cracking behavior, and failure mechanisms. Recent advancements have involved the incorporation of cohesive zone modeling and bond–slip relationships to more realistically capture the interaction between FRP and concrete under various loading conditions [40,47,48,49,50]. Notably, Haryanto et al. [42] investigated NSM-strengthened T-beams using cohesive zone modeling, revealing that variations in CFRP depth of embedment significantly govern global response through their effect on bond performance. Building on these developments, Nugroho et al. [47] carried out numerical investigations of the behavior of NSM-CFRP systems used to strengthen prestressed concrete T-beams under monotonic loading, with a particular focus on the negative moment region. Their FE model captured the nonlinear behavior of the concrete, steel, and prestressing strands, while CFRP rods and sheets were idealized as having linear elasticity. A cohesive interface model was employed to replicate the bond behavior between the CFRP and concrete. The model was validated against prior experimental benchmarks and yielded high predictive accuracy. Parametric analyses revealed that for a low level of initial prestressing, half-embedded CFRP rods improved the ultimate load capacity by 11.20%, while full embedment yielded a 14.02% gain. Under a high level of prestressing, the enhancements were 15.76% and 16.36%, respectively. Furthermore, all strengthened configurations showed reduced tensile and compressive damage in the concrete, thereby affirming the effectiveness of both embedment strategies.

Despite the demonstrated benefits of NSM-CFRP systems, their seismic performance, especially in prestressed members with varying embedment depths, remains insufficiently explored. Previous works on hybrid structural systems and joint performance have highlighted the importance of accurate modeling and experimental validation for assessing seismic resilience. In addition, studies on FRP–concrete interfaces have shown that bond behavior and associated failure mechanisms are decisive factors governing the overall effectiveness of strengthening under seismic demand. For example, investigations on precast prestressed steel-reinforced concrete (SRC) composite frames with engineered cementitious composite (ECC) materials have demonstrated that such systems can improve energy dissipation, ductility, and damage resistance under cyclic loading [51], while other studies have proposed simplified yet effective tools for beam–column joint design that ensure code compliance and structural reliability even at early design stages [52].

To address this gap, the present study extends the previous work of Nugroho et al. [47], which was limited to monotonic loading, by employing FE modeling to investigate the flexural behavior of NSM-CFRP-strengthened prestressed T-beams under seismic loading. Unlike earlier studies, this research incorporates reversed cyclic, near-fault, and far-fault ground motion scenarios, allowing for a more comprehensive assessment of seismic performance. The developed models were first validated against experimental data from RC T-beams, prestressed I-beams, and slabs to ensure simulation reliability. A subsequent parametric study examined the effects of CFRP embedment depth and initial prestress level under the different seismic scenarios. The results confirmed that even half-embedded CFRP rods can significantly improve structural performance under repeated loading, offering a practical alternative when full-depth installation is not feasible due to construction or design constraints. This research contributes to a deeper understanding of NSM system behavior under seismic demand and supports the advancement of resilient and adaptable retrofitting strategies for prestressed concrete infrastructure.

## 2. Novelty of the Research

The novelty of this study lies in extending research on NSM-CFRP strengthening in the negative moment region of RC T-beams from monotonic loading to seismic scenarios, including reversed cyclic, near-fault, and far-field excitations. The work provides a direct comparison between half and full embedment in prestressed members, addressing the practical feasibility of partial embedment where full-depth installation is limited. The study also presents a validated finite element framework capable of capturing both global and local responses, offering a reliable basis for parametric studies and the development of performance-based retrofit strategies for prestressed concrete structures.

## 3. Specimen Configuration

**Figure 1:**
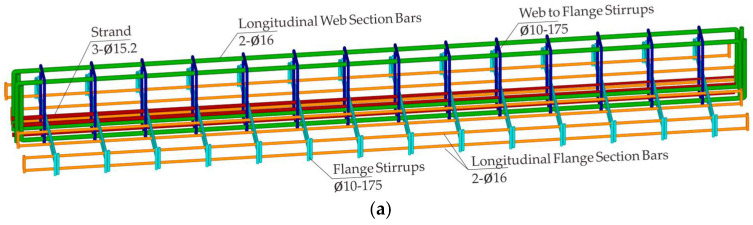

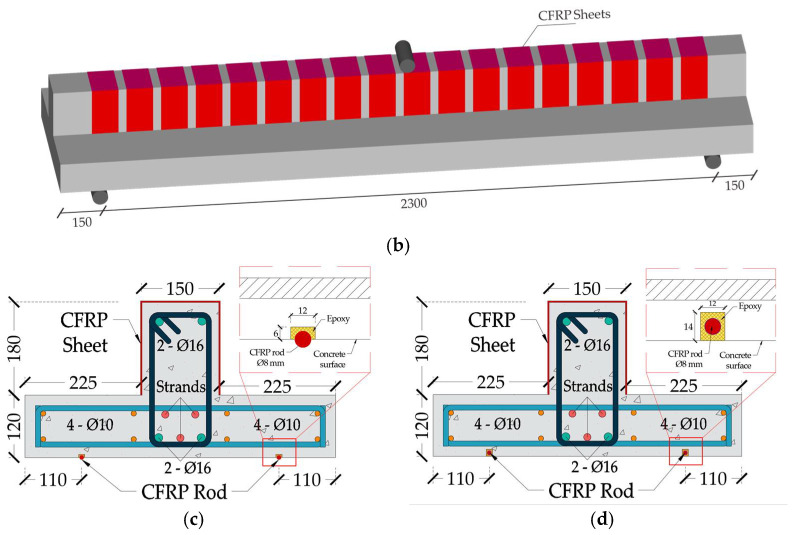
Configuration of the prestressed concrete T-beam and applied strengthening schemes (all dimensions in mm): (**a**) overall beam geometry with reinforcement layout; (**b**) strengthening arrangement with NSM-CFRP rods; (**c**) section illustrating half-embedded CFRP rods (BH specimen); (**d**) section illustrating fully embedded CFRP rods (BF specimen).

Building upon the foundational experimental work conducted by Haryanto et al. [41,42], our numerical investigation explores the physical characteristics of prestressed concrete T-beams retrofitted with CFRP rods under seismic loading. To ensure consistency and enable direct comparability, the geometric configurations, material properties, and strengthening schemes adopted in the simulation closely replicate those used in the referenced experimental study. The modeling program includes three beam configurations: an unstrengthened control specimen (BN) and two strengthened variants incorporating different CFRP retrofitting schemes. Figure 1 illustrates the geometric layout and strengthening arrangement.

Each beam specimen measured 2600 mm in total length and had a cross-sectional profile characterized by a 300 mm overall depth, a 150 mm web width, a 600 mm flange width, and a 120 mm flange thickness. The primary longitudinal reinforcement system comprised two 16 mm diameter deformed steel bars positioned symmetrically within the tensile and compressive zones. Supplementary longitudinal reinforcement within the flange consisted of eight plain round bars, each with a 10 mm diameter. Shear reinforcement was provided by closed steel stirrups 10 mm in diameter, uniformly distributed with a 175 mm center-to-center spacing along both the web and flange regions.

The two strengthened beam specimens were created with the NSM technique, wherein a pair of CFRP rods 8 mm in diameter was embedded longitudinally within the concrete flange to replicate the tensile zone of the negative moment region. Following a standard 28-day curing period, shallow surface grooves were precision-cut into the concrete cover to accommodate the CFRP installation. A high-strength epoxy adhesive was applied to ensure effective bond development between the CFRP rods and the surrounding concrete matrix. To enhance the shear resistance, additional confinement was provided through the application of discrete U-shaped CFRP strips, each 100 mm in width, which were bonded externally to the web surfaces with epoxy. These U-wraps were deployed at uniform 130 mm intervals along the full length of the beam span.

A notable contribution of this investigation lies in the evaluation of a nonconventional CFRP strengthening strategy involving partial embedment of reinforcement in the negative moment region of prestressed concrete T-beams. Unlike standard NSM techniques, which involve full-depth embedment, as prescribed in ACI 440 [36], this study presents and analyzes an alternative configuration that is intended for scenarios where achieving full groove depth is not feasible due to field constraints. Two retrofitted beam specimens were developed: BF, with CFRP rods fully embedded according to code provisions, and BH, with rods embedded to only half their diameter and intentionally left without a cover layer. These strengthened beams were assessed alongside an unstrengthened reference specimen to determine the structural consequences of reduced embedment depth.

To adapt the original RC T-beam configuration to prestressed applications, internal low-relaxation tendons were introduced to simulate the behavior of a prestressed concrete section. The revised design incorporated three prestressing strands that were symmetrically positioned within the flange region. The tendon layout and associated material properties were based on the specifications reported by Zhao et al. [11], in which each strand consisted of four-wire bundles with a nominal diameter of 15.2 mm and an ultimate tensile capacity of 1860 MPa. Two distinct levels of prestress were introduced in the analysis to capture varying structural responses: a lower level corresponding to 35% of the strand’s ultimate strength, and a higher level corresponding to 70%. Table 1 summarizes the specimen configurations and design parameters.

**Figure 2:**
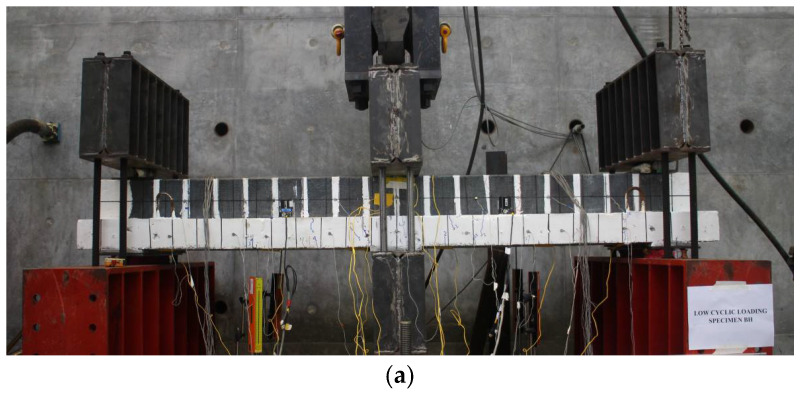

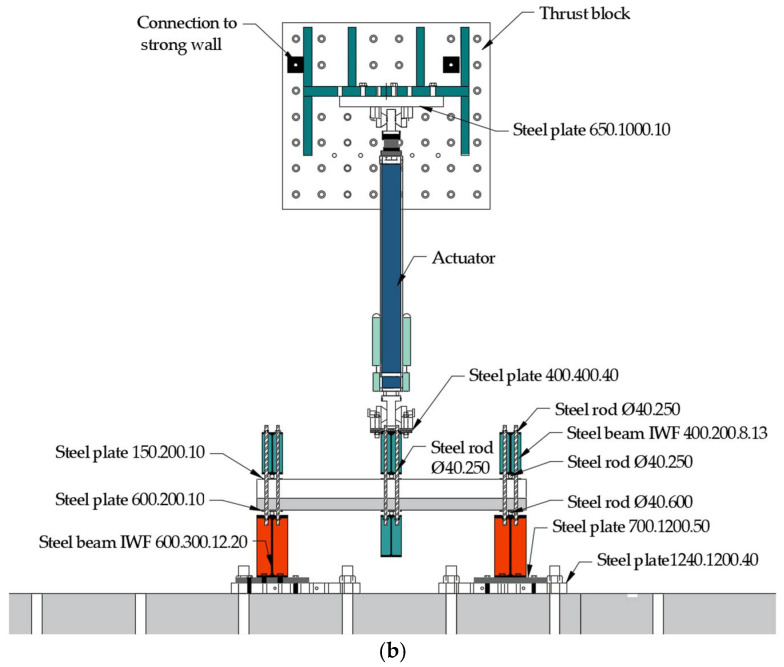
Test setup adopted by Haryanto et al. [41]: (**a**) photograph; (**b**) schematic.

Consistent with the methodology adopted by Haryanto et al. [41], as shown in Figure 2, the prestressed concrete T-beams were inverted to simulate negative flexural conditions within the slab region, with the span between supports set at 2300 mm. The test specimens were subjected to quasi-static reversed cyclic loading under a three-point bending configuration, which was conducted on the rigid strong floor of the laboratory. A dynamic hydraulic actuator, rated at 500 kN capacity and anchored to a vertical reaction wall, was used to apply the cyclic load in the downward direction. To ensure consistent boundary conditions while enabling bidirectional loading, the beams were secured using clamping assemblies composed of steel I-section profiles positioned at both the midspan and near the support zones. A roller interface was placed between each clamp and the beam surface to allow for controlled rotation and effective load transfer. This setup permitted the actuator to deliver both tensile (uplift) and compressive (downward) cycles along a symmetric loading path, effectively reproducing the cyclic flexural demands associated with seismic excitation under quasi-static conditions.

**Figure 3:**
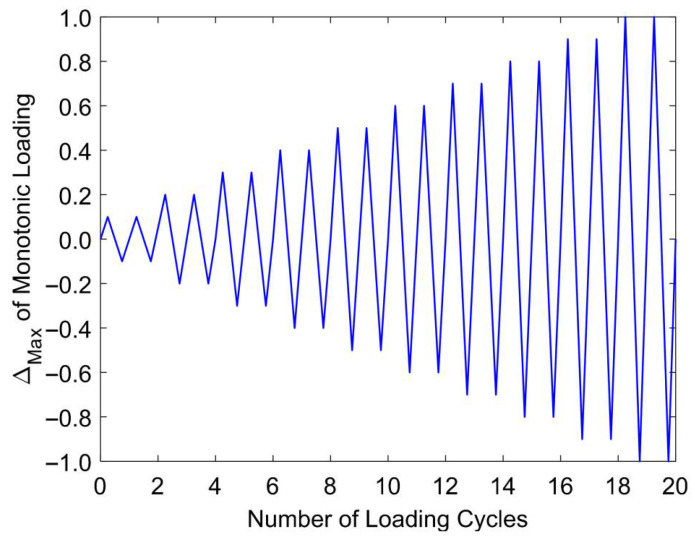
Loading protocol adopted by Haryanto et al. [41].

All specimens were subjected to displacement-controlled reversed cyclic loading, with each level of displacement applied over two consecutive cycles to capture hysteretic behavior and degradation trends. The amplitude of the imposed displacements was increased incrementally by 10% of the peak displacement observed in the control beam under monotonic loading, based on benchmark results from a prior investigation [41]. The loading sequence, illustrated in Figure 3, was executed at a constant actuator velocity of 4 × 10^−4^ m/s, a rate classified as being within the low-rate loading regime. It should be noted that this configuration represents vertical cyclic loading applied at midspan in a three-point bending setup, which generates alternating tension and compression zones in the beam section to simulate the flexural demands typically induced by seismic ground shaking.

## 4. Development of the FE Model

This section outlines the development of a three-dimensional FE model aimed at replicating the structural response of prestressed concrete T-beams retrofitted with NSM-CFRP rods in the negative flexural region under seismic loading. Numerical simulations were performed using the ABAQUS (Version 6.14) commercial software package [53], which was selected for its robust capabilities in capturing the nonlinear behavior of reinforced concrete components, as demonstrated in previous studies [11,40].

### 4.1. Geometry and Element Description

To enhance computational efficiency while maintaining model fidelity, the FE simulation was limited to one-quarter of the prestressed concrete beam by exploiting geometric, loading, and boundary symmetries. Zheng et al. [54] reported that such model reduction exerts minimal influence on global and local responses (e.g., load–deflection, stiffness, and stress distribution) while substantially decreasing computation time, thereby supporting the validity of the adopted strategy. The numerical assembly comprised several interacting components, including the concrete substrate, internal reinforcement, prestressing tendons, NSM-CFRP rods, epoxy adhesive, and externally bonded CFRP sheets.

The solid components, namely the concrete, epoxy, and CFRP rods, were discretized using eight-node hexahedral elements with reduced integration (C3D8R). This element type was selected because the reduced integration scheme (one integration point per element) significantly decreases computational demand while remaining well suited for large deformation and nonlinear material behavior, as it alleviates shear locking compared to fully integrated elements. Both the internal steel reinforcement and prestressing strands were modeled as linear 3D truss elements (T3D2) to reflect their primarily axial force-resisting behavior.

**Figure 4:**
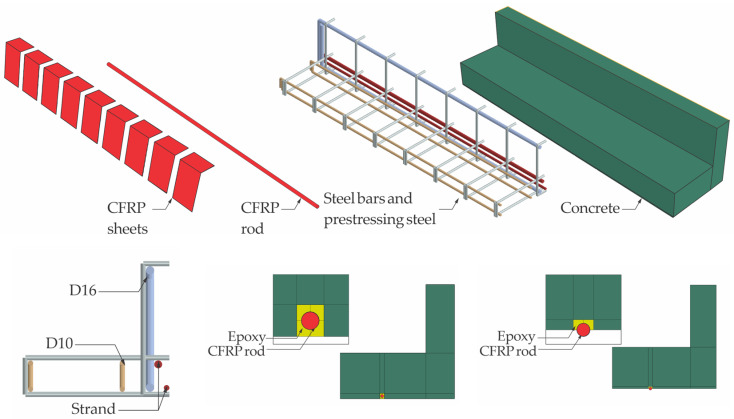
Structural geometry and section configuration adopted in the numerical model [47].

**Figure 5:**
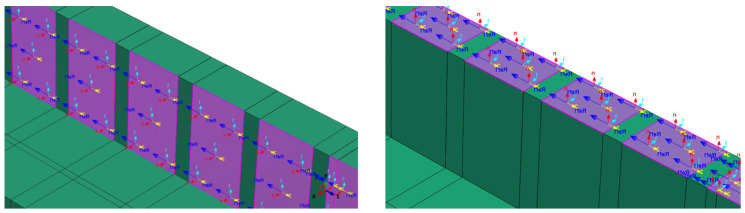
Fiber orientation of the CFRP sheet [47].

The CFRP sheets were modeled using conventional shell techniques and applied directly to the concrete surface through the skin function within the modeling environment. In this approach, the shell elements representing the CFRP laminates were tied to the outer surface of the solid concrete elements, enforcing compatibility of nodal displacements across the interface. This ensured full adhesion between the two materials, thereby preventing slip or separation during loading and allowing stresses to be transferred consistently from the CFRP sheets into the concrete substrate. The sheets were meshed using four-node, reduced-integration shell elements with finite membrane strains (S4R). The composite layup was defined by explicitly specifying the material stiffness, ply thickness, number of layers, and fiber orientation to accurately capture the anisotropic behavior of the CFRP system. Figure 4 presents the details of the geometric configuration and sectional properties of the model. The fiber directions, shown in Figure 5, correspond to the principal material axes, where the 1-direction and 2-direction represent the longitudinal and transverse orientations, respectively.

In addition, the mesh density was verified through a convergence analysis to ensure that the selected discretization did not compromise accuracy. A control beam subjected to monotonic loading was analyzed using three mesh sizes (25 mm, 26 mm, and 27 mm), and the predicted load–deflection responses were compared with experimental results.

**Figure 6:**
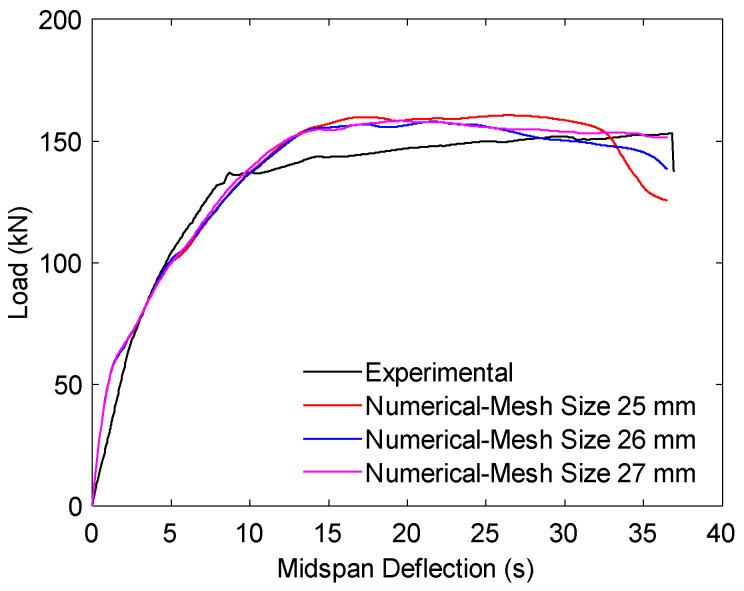
Mesh sensitivity analysis of the control beam under monotonic loading.

As shown in Figure 6, the 26 mm mesh size provided the closest agreement while maintaining computational efficiency. Accordingly, this mesh size was adopted for all subsequent simulations under seismic loading to ensure reliable predictions of crack initiation, debonding, and overall structural response.

### 4.2. Constitutive Models of Materials

#### 4.2.1. Concrete

To accurately replicate the mechanical behavior of concrete under both compressive and tensile loading conditions, a two-phase material modeling approach was adopted. The initial phase captured the elastic response, which was governed by fundamental material constants including the Young’s modulus and Poisson’s ratio. In this context, Poisson’s ratio was taken as 0.2, while the elastic modulus, $E_{c}$ , was computed in accordance with the provisions of ACI 318-19 [55], with an empirical adjustment factor derived from the Taiwanese concrete code [56] to reflect the regional material properties. The formulation used to determine the modulus of elasticity was as follows:

(1) $$E_{c}=0.8\times 4700\sqrt{f_{c}^{\prime }}$$

Beyond the elastic threshold, the material model changed from a linear to a nonlinear stress–strain formulation to more accurately capture the inelastic response of concrete. Under compressive loading, this nonlinear regime is characterized using the constitutive framework proposed by Hsu and Hsu [57], as defined by Equations (2)–(4). The model describes the post-yield behavior commencing at $0.5f_{c}^{\prime }$ , extending through the softening branch, and continuing until the stress diminishes to $0.3f_{c}^{\prime }$ , thereby encapsulating both the peak and degradation phases of the compressive response.

(2) $$f_{c}=\left(\frac{\beta \left(\varepsilon_{c}/\varepsilon_{0}\right)}{\beta -1+{\left(\varepsilon_{c}/\varepsilon_{0}\right)}^{\beta }}\right)f_{c}^{\prime }$$

(3) $$\beta =\frac{1}{1-\left(f_{c}^{\prime }/\varepsilon_{0}E_{c}\right)}$$

(4) $$\varepsilon_{0}=8.9\times 10^{-5}f_{c}^{\prime }+2.144\times 10^{3}$$

where $f_{c}\;$ denotes the compressive stress corresponding to a given concrete strain $\varepsilon_{c}$ ; β represents a dimensionless material coefficient that governs the curvature of the stress–strain response; $f_{c}^{\prime }\;$ is the peak compressive strength of concrete; and $\varepsilon_{0}$ represents the strain associated with $f_{c}^{\prime }$ . For consistency in interpretation, all stress values are expressed in units of kips per square inch (ksi), where 1 MPa is equivalent to 0.145037743 ksi.

In the tensile regime, once the concrete surpasses its cracking threshold, the material response transitions into a post-peak softening phase. This tensile degradation was captured using the tension stiffening model proposed by Alfarah et al. [58], which accounts for the gradual reduction in tensile stress following cracking. The relationship governing this behavior is expressed through the normalized stress ratio $f_{t}/f_{t}^{\prime }$ , where $f_{t}$ denotes the current tensile stress and $f_{t}^{\prime }$ represents the peak tensile capacity. The functional form of this relation is given in Equation (5).

(5) $$\frac{f_{t}}{f_{t}^{\prime }}=\left[1+{\left(C_{1}\frac{w}{w_{c}}\right)}^{3}\right]e^{-c_{2}\frac{w}{w_{c}}}-\frac{w}{w_{c}}\left(1+C_{1}^{3}\right)e^{-c_{2}}$$

In the above formulation, the maximum tensile strength $f_{t}^{\prime }$ is estimated using the empirical relation $f_{t}^{\prime }=0.33\sqrt{f_{c}^{\prime }}$ , in accordance with the ACI 318-19 recommendations [55]. The model incorporates two empirical constants, $C_{1}=3.00$ and $C_{2}=6.93$ , as proposed by Hordijk [59], to define the shape of the post-cracking softening curve. The parameter $w_{c}$ represents the critical crack opening displacement and is determined as a function of the concrete’s tensile strength and fracture energy $G_{F}$ per unit area [59], as expressed in Equation (6). The fracture energy $G_{F}$ , which governs the tensile softening behavior of concrete, is estimated using the empirical expression provided in Equation (7) [60].

(6) $$w_{c}=5.14\frac{G_{F}}{f_{t}^{\prime }}$$

(7) $$G_{F}=0.073{\left(f_{t}^{\prime }\right)}^{0.18}$$

The tensile strain ( $\varepsilon_{t}$ ) within the post-peak softening regime is evaluated by relating it to the strain at peak tensile stress ( $\varepsilon_{t}^{\prime }$ ), crack opening ( w ) and mesh size coefficient ( $I_{eq}$ ). This formulation assumes that each element accommodates a single discrete crack [58] and is expressed in Equation (8).

(8) $$\varepsilon_{t}=\varepsilon_{t}^{\prime }+\frac{w}{I_{eq}}$$

**Figure 7:**
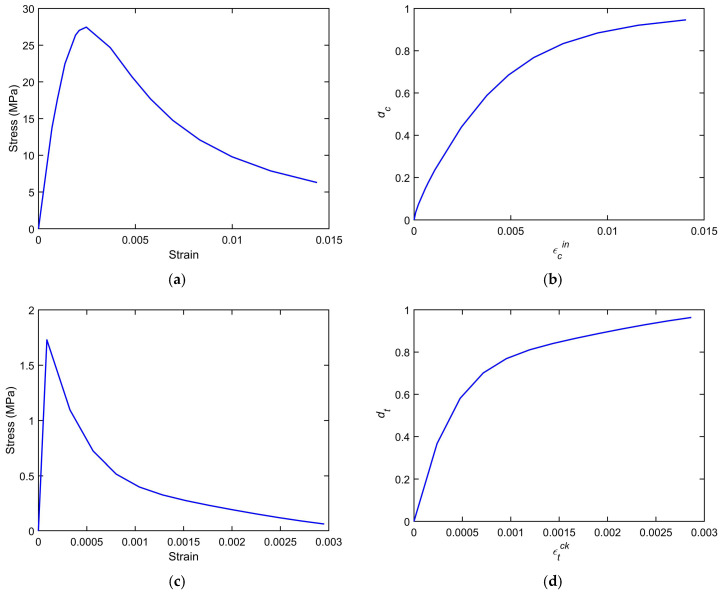
Concrete material response adopted in FE modeling: (**a**) uniaxial compression stress–strain curve; (**b**) evolution of compressive damage; (**c**) uniaxial tension stress–strain curve; (**d**) evolution of tensile damage.

Using an average compressive strength of 27.12 MPa, a value obtained from prior experimental characterization [41,42], the corresponding stress–strain curves employed in the simulation were created as shown in Figure 7a,c. To capture the inelastic behavior of concrete, the Concrete Damage Plasticity (CDP) model was adopted, which has been widely implemented in ABAQUS to model reinforced concrete structures [53]. The CDP model, originally formulated by Lubliner et al. [61] and refined in subsequent studies [62,63,64], incorporates a unified plastic-damage framework that accounts for both tensile and compressive mechanisms of failure. It combines isotropic plasticity with scalar degradation variables representing damage-induced stiffness reduction, thereby providing a robust constitutive formulation that is capable of simulating the progressive degradation and nonlinear response of concrete under multi-axial loading conditions.

Concrete failure is characterized by two principal modes of degradation: tensile-induced cracking and compressive crushing [65]. These phenomena are quantified using scalar damage variables, *d_t_* and *d_c_*, with values ranging from zero (representing intact material) to one (indicating complete material degradation). In the present study, these damage indices were computed using the formula in Equation (9), which has been previously calibrated and validated for RC simulations by Wei et al. [66].

(9) $$d_{c}=\frac{(1-\eta_{c}){\tilde{\varepsilon }}_{c}^{in}E_{0}}{f_{c}+(1-\eta_{c}){\tilde{\varepsilon }}_{c}^{in}E_{0}}\;\;\;;\;\;\;d_{t}=\frac{(1-\eta_{t}){\tilde{\varepsilon }}_{t}^{ck}E_{0}}{f_{t}+(1-\eta_{t}){\tilde{\varepsilon }}_{t}^{ck}E_{0}}$$

where parameters $\eta_{c\;}$ and $\eta_{t\;}$ represent the ratios between the plastic strain and inelastic strain in compression, and cracking strain in tension, respectively, with recommended values of 0.60 and 0.90 [66].

In addition to the stress–strain laws, the CDP formulation requires the definition of several plasticity parameters, namely the dilation angle ( ψ ), flow potential eccentricity (*e*), the biaxial-to-uniaxial compressive strength ratio ( $\sigma_{b0}/\sigma_{c0}$ ), the deviatoric surface shape factor ( $K_{c}$ ), and the viscosity parameter. Default or recommended values were adopted for ψ , *e*, and $K_{c}$ , while the viscosity parameter was calibrated based on prior studies [67]. The selected parameters are summarized in Table 2.

The evolution of compressive and tensile damage within the concrete domain is graphically represented in Figure 7b and Figure 7d, respectively, where the distinct degradation paths associated with each failure mode can be observed.

#### 4.2.2. Steel Rebar and Prestressing Strand

The constitutive behavior of the steel reinforcement was modeled using a bilinear stress–strain relationship that included strain hardening. The elastic modulus ( $E_{s}$ ) and Poisson’s ratio were specified as 200 GPa and 0.30, respectively, while all remaining mechanical properties were derived from experimental testing data reported by Haryanto et al. [41,42]. For the 10 mm diameter bars, the material had a yield strength of 350.67 MPa, an ultimate tensile strength of 500.85 MPa, and a corresponding ultimate strain of 0.1311. In contrast, the 16 mm diameter bars had a higher yield strength of 492.5 MPa, an ultimate strength of 727.51 MPa, and an ultimate strain of 0.0817. These values reflect the distinct stress–strain characteristics associated with different bar diameters and were incorporated into the numerical model accordingly.

**Figure 8:**
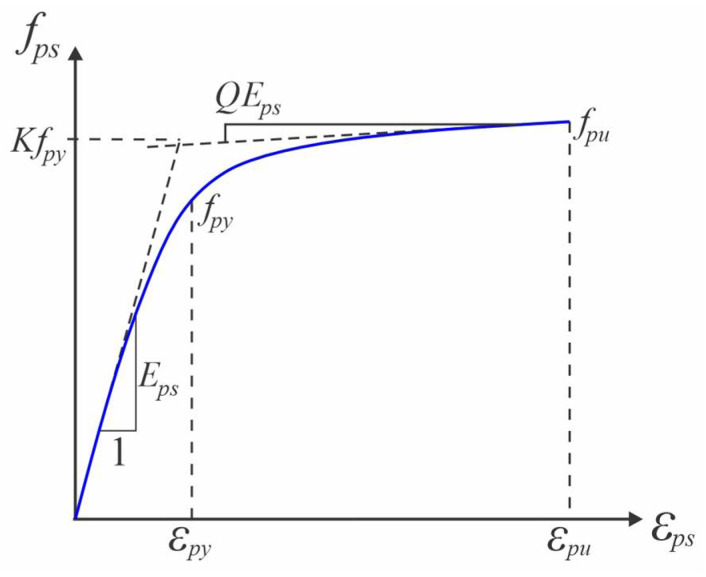
Stress–strain relationship for the prestressing strand.

The mechanical properties of the prestressing strand were defined by a Young’s modulus of 195 GPa and a Poisson’s ratio of 0.30. The material exhibited a yield strength of 1675 MPa, with an ultimate tensile strength of 1860 MPa and an ultimate strain capacity of 0.035, as reported by Zhao et al. [11]. To capture the nonlinear stress–strain response under high-stress conditions, the behavior of the strand was modeled using the Menegotto–Pinto formulation [68], a widely adopted expression for the cyclic behavior of steel. This constitutive relationship is graphically represented in Figure 8 and given mathematically in Equations (10) and (11).

(10) $$f_{ps}=E_{ps}\varepsilon_{ps}\left\{Q+\frac{1-Q}{{\left[1+{\left(\frac{E_{ps}\varepsilon_{ps}}{Kf_{py}}\right)}^{N}\right]}^{1/N}}\right\}$$

(11) $$Q=\frac{f_{pu}-Kf_{py}}{E_{ps}\varepsilon_{pu}-Kf_{py}}$$

where $E_{ps}\;$ denotes the modulus of elasticity, $f_{py}$ corresponds to the yield strength, and $f_{pu}\;$ represents the ultimate tensile strength of the prestressing steel. The parameters *N*, *K*, and *Q* are model coefficients that are calibrated from the experimentally observed stress–strain behavior and the characteristic shape of the material response curve. In situations where detailed stress–strain data are unavailable, standard values of *N* = 7.00 and *K* = 1.00 may be adopted for typical prestressing steels, as recommended by Naaman [69].

#### 4.2.3. CFRP Composites

In this investigation, two forms of CFRP reinforcement were considered: rods and externally bonded sheets. The CFRP rods were modeled based on the assumption of a linear elastic–brittle response, which was characterized by a proportional stress–strain relationship up to rupture, with no yield plateau or post-yield deformation. This idealized behavior reflects the intrinsic properties of CFRP, which fails abruptly once its tensile capacity is exceeded. The properties of the rod specimens, identified as Sika^®^ CarboDur^®^ BC and supplied by PT. SIKA Indonesia (Bogor, Indonesia), were defined based on manufacturer-provided specifications [70]. The key mechanical parameters were a nominal diameter of 8 mm, a tensile strength of 3100 MPa, a modulus of elasticity of 148 GPa, and an ultimate tensile strain of 0.021.

The mechanical properties of the CFRP sheet system used for shear strengthening are summarized in Table 3. According to the manufacturer’s datasheet, key properties include the tensile strength in the principal fiber direction $X_{1}^{T}\;$ and the corresponding longitudinal elastic modulus $E_{11}$ . Additional parameters required for complete orthotropic material definition were estimated following the specifications outlined in TB-06-CRP-1 [71]. For simulation purposes, the CFRP sheets were modeled as laminated orthotropic layers to capture their anisotropic elastic behavior. Failure was governed by the Hashin damage initiation criterion [72], and a Poisson’s ratio of 0.30 was adopted to represent the lateral strain response under uniaxial loading.

### 4.3. Boundary Conditions

**Figure 9:**
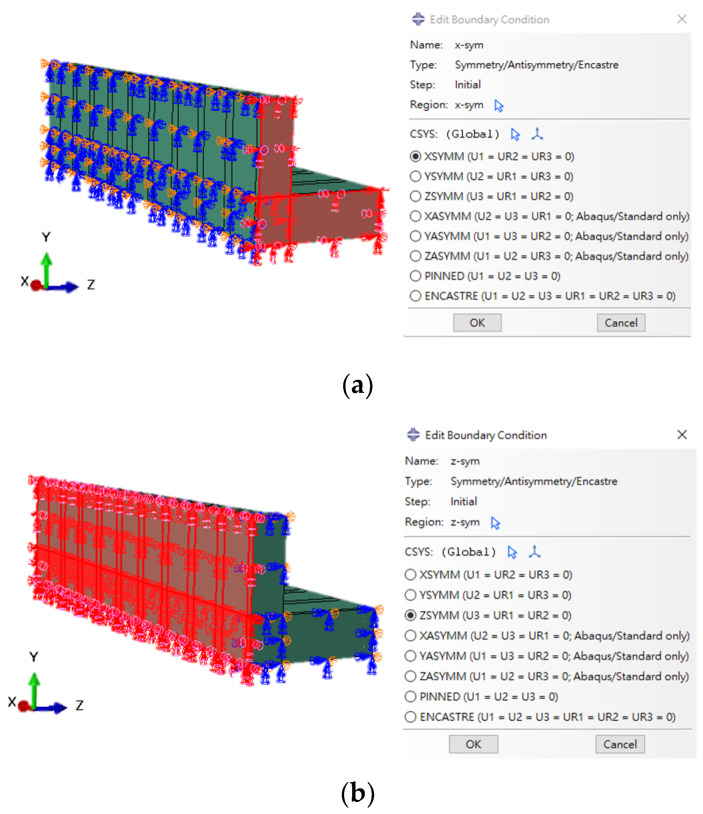
Symmetry conditions: (**a**) y-z plane; (**b**) x-y plane.

Symmetry was imposed on the longitudinal cut plane by restricting out-of-plane displacement, as shown in Figure 9, thereby allowing for computational efficiency while preserving mechanical fidelity in terms of load transfer and deformation response.

To replicate the cyclic loading protocol employed in the experimental setup, the boundary conditions for the FE model were configured to reflect the inverted three-point bending arrangement, thereby generating negative flexural stresses in the slab region. The bottom flange at one support was constrained in the vertical direction to simulate a simple support, while prescribed midspan displacements were applied at the opposite end through a reference point to emulate the actuator motion. The analysis was carried out using a quasi-static procedure capable of capturing load reversals, unloading–reloading behavior, and nonlinear material responses.

### 4.4. Contact Interaction

To streamline the simulation and reduce the computational overhead, the internal steel reinforcements were assumed to be perfectly bonded to the surrounding concrete matrix, with any potential slip at the steel–concrete interface being neglected. This idealized condition reflects a fully embedded interaction with continuous strain compatibility. Likewise, no explicit contact interface was defined between the externally bonded CFRP sheets and the concrete substrate; instead, the CFRP laminates were implemented as skin elements directly attached to the concrete surface, thus inherently enforcing full adhesion and eliminating the need for interface modeling.

For the CFRP rods, a different strategy was adopted to reflect the distinct bonding mechanism. In the case of fully embedded specimens, the interface between the CFRP rods and concrete was modeled indirectly through an intermediate epoxy layer, which functioned as the bonding medium. Tie constraints were applied to both the epoxy–concrete and epoxy–CFRP interfaces to enforce perfect bond conditions and prevent relative slip. In the FE framework, a tie constraint links the degrees of freedom of the slave surface nodes (epoxy or CFRP) to those of the master surface nodes (concrete), thereby ensuring full displacement compatibility and transferring stresses across the interface without separation. This modeling simplification was supported by experimental evidence indicating strong adhesive performance and the absence of epoxy failure, thus justifying the assumption from both practical and computational perspectives.

In contrast, for half-embedded configurations, where experimental results indicated debonding behavior at the rod–concrete interface, a more detailed interaction model was employed. A surface-based cohesive zone model was used to simulate the bond-slip response, which included a linear elastic traction–separation law formulated within an uncoupled framework. The model utilized three orthogonal stiffness parameters, in the normal and two shear directions, with values adopted from validated studies on half-embedded CFRP rods [39], as listed in Table 4.

To capture the post-peak behavior, a damage evolution law based on fracture energy was introduced [47], in which the area under the stress–displacement curve corresponds to the material’s fracture energy, ensuring energy-consistent softening; a linear softening profile was adopted to represent the progressive loss of stiffness following damage initiation.

### 4.5. Predefined Field

The initial conditions within the FE model were set using a predefined field, which enabled the simulation of prestress effects prior to external loading. Two levels of prestressing were assigned to the strands, corresponding to 35% (651 MPa) and 70% (1302 MPa) of their ultimate tensile capacity. These values were selected to represent low and high prestress conditions typically used in practice and are consistent with ranges reported in previous experimental and numerical studies on prestressed concrete members [2,3,4]. In addition, a uniform gravitational load was applied over the beam’s entire geometry to account for self-weight. To ensure that the internal stress state prior to service loading was accurately represented, the prestress force was incrementally introduced in a staged manner, thus ensuring proper stress distribution before the application of external seismic demands.

## 5. Results and Discussion

### 5.1. Assessment of Numerical Model Accuracy

The accuracy of the FE formulation developed for prestressed concrete members retrofitted with NSM-CFRP rods and externally bonded sheets was assessed through a multi-stage validation strategy involving three benchmark cases. In the first stage, the focus was on replicating the experimental behavior of CFRP-strengthened RC T-beams, as described by Haryanto et al. [41]. It was then tested on a prestressed concrete I-beam from the work of Rabczuk and Eibl [73] to assess its ability to reproduce flexural behavior governed primarily by prestressing. Finally, validation was extended to a CFRP-strengthened prestressed concrete slab based on the experimental program of Shahawy et al. [74]. These validation exercises were considered to demonstrate the robustness and predictive fidelity of the FE model across various structural typologies and reinforcement configurations involving CFRP-based strengthening.

#### 5.1.1. RC T-Beams Strengthened with CFRP Subjected to Cyclic Loading

The initial validation scenario was based on an experimental investigation of RC T-beams, without prestressing, retrofitted using NSM-CFRP rods in combination with externally bonded CFRP sheets, as documented by Haryanto et al. [41]. The geometric configuration, reinforcement detailing, and strengthening layout corresponding to this case were as described in Section 2. The finite element model was developed to replicate the experimental conditions with high fidelity, including consistent boundary constraints, loading protocols, and CFRP application techniques, to ensure a meaningful comparison between the numerical and experimental outcomes.

The material properties adopted in this validation case reflect those reported in the corresponding experimental study. The concrete had a compressive strength ( $f_{c}^{\prime }$ ) of 27.12 MPa. The 10 mm plain round bars used for internal reinforcement had a yield strength $\left(f_{y}\right)$ of 350 MPa and an ultimate tensile strength $\left(f_{u}\right)$ of 500 MPa. For the 16 mm deformed bars, the yield and ultimate strengths were 492 MPa and 727 MPa, respectively. The NSM CFRP rods were characterized by a tensile strength of 3100 MPa and an elastic modulus of 148 GPa. The externally bonded CFRP sheets had a nominal thickness of 0.131 mm.

To optimize computational efficiency, only one-quarter of the beam specimen was modeled by applying symmetric boundary conditions along two orthogonal planes. The concrete and CFRP rods were meshed using C3D8R reduced-integration brick elements, while the internal steel reinforcement was represented using T3D2 truss elements to simulate axial load transfer. The CFRP sheet was modeled as an S4R shell element and applied as a skin layer directly onto the concrete surface, assuming a perfect bond with no relative slip. In the fully embedded configuration (BF), tie constraints were used to simulate perfect adhesion between the epoxy, concrete, and CFRP rods, reflecting experimental evidence of intact bonding. Conversely, for the half-embedded configuration (BH), a cohesive surface interaction model was implemented at the epoxy–CFRP interface to account for the debonding behavior observed during testing. The stiffness parameters for the cohesive interface in the BH model are provided in Table 4.

**Figure 10:**
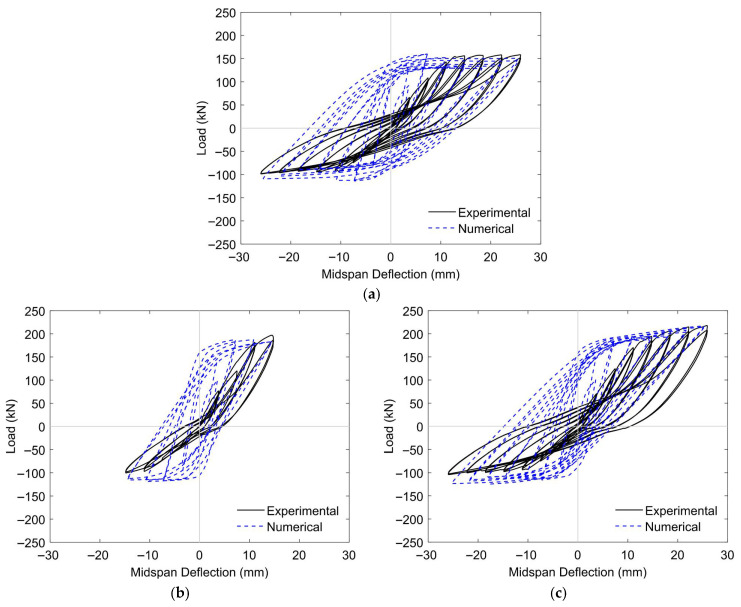
Experimental [41] and numerical hysteresis curves: (**a**) BN; (**b**) BH; (**c**) BF.

Table 5 presents a comparative summary of the ultimate load capacities and midspan deflections obtained from both experimental testing and FE simulations for the evaluated beam specimens. Corresponding hysteresis curves for three representative configurations are shown in Figure 10. The consistency between the numerical predictions and experimental observations indicated a high level of fidelity for the model. In quantitative terms, the FE model was able to predict the ultimate load and associated midspan deflection within error margins ranging from 1.36% to 7.09% and 0.17% to 2.34%, respectively, thereby confirming the model’s reliability in capturing both the strength and deformation characteristics of the tested systems.

To further assess the predictive quality of the FE simulations, statistical performance indicators, specifically the normalized mean square error (NMSE), were employed to quantify the level of agreement between the simulated and experimental responses [75,76,77,78]. The computed value of 0.0025 for the NMSE reflects a high degree of numerical accuracy and suggests that the model’s predictive capability for load–deflection behavior is within acceptable limits.

However, while the FE models successfully replicated the overall loading process, they were unable to capture certain critical features observed experimentally, most notably the absence of pinching in the numerical hysteresis curves. This limitation is likely related to the characteristics of the Concrete Damage Plasticity (CDP) model, which, although effective in simulating global cracking and crushing, does not fully account for progressive stiffness degradation and the crack closure–reopening mechanisms that govern pinching behavior. In addition, the assumption of perfect adhesion between concrete and steel reinforcement, as well as between epoxy and CFRP rods in the full-embedment configuration, neglects local slip, partial debonding, and frictional effects that typically contribute to hysteretic degradation. The material models for CFRP and steel also did not incorporate cyclic deterioration or fatigue-type damage accumulation, which may have led to the overestimation of energy dissipation. As a result of these combined limitations, the simulated hysteresis loops appeared “fuller” than those obtained experimentally, reflecting reduced stiffness degradation and exaggerated energy absorption in the numerical predictions.

**Figure 11:**
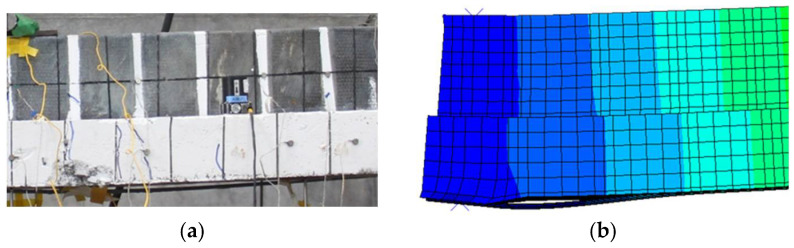
Comparison of CFRP rod debonding from (**a**) experimental observation [41] and (**b**) numerical model for the BH beam. In the numerical model, the color contour represents scaled displacement, where darker blue indicates the maximum displacement.

In a prior study conducted by Haryanto et al. [41], the unstrengthened specimen (BN) underwent a flexural-shear mode of failure, initiated by crack formation at the bottom surface of the beam under tensile flexural stress. Subsequent crack propagation followed an inclined trajectory, widening and extending upward toward the compression zone. In contrast, for the CFRP-strengthened specimens (BH and BF), prominent flexural cracks were observed along the tension face and were predominantly concentrated in the midspan region. Notably, in the BH specimen, partial debonding of the CFRP rod occurred at the site of major cracking, an effect accurately captured by the FE simulation, as illustrated in Figure 11. Ultimately, both strengthened configurations failed via a flexural mode, governed by the propagation of dominant midspan cracks that led to a loss of load-carrying capacity.

#### 5.1.2. Prestressed Concrete I-Beam

The second validation scenario involved simulation of the behavior of prestressed concrete, with the finite element model calibrated against the experimental results reported by Rabczuk and Eibl [73], who examined prestressed beams featuring I-shaped cross-sections. This case was selected to assess the model’s ability to accurately represent the effects of the prestressing force within the structural system. The test specimen incorporated two prestressing tendons, each 12 mm in diameter, embedded within the lower flange and tensioned to a force of 80 kN per strand. In addition, a single non-prestressed longitudinal reinforcement bar, 10 mm in diameter, was placed in the upper region of the cross-section to complete the internal reinforcement layout.

**Figure 12:**
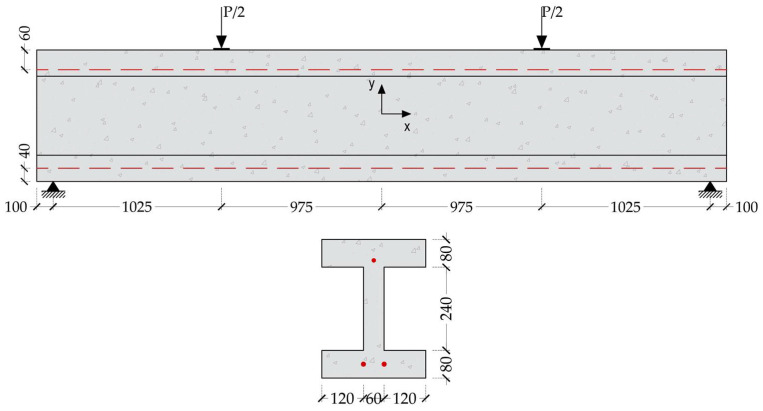
Specimen configuration in the study of Rabczuk and Eibl [73] (dimensions in mm). The dashed red lines denote the longitudinal reinforcement and prestressing tendons, while the red dots indicate their cross-sectional positions.

The prestressing tendons used in the validation specimen had a yield strength of 1420 MPa, an ultimate tensile strength of 1570 MPa, a Young’s modulus of 195 GPa, and a material density of 7.8 × 10^−3^ g/mm^3^. The concrete mix had a uniaxial compressive strength ( $f_{c}^{\prime }$ ) of 45 MPa. Following the application of prestressing, the beam was tested under a four-point bending configuration using monotonic static loading up to failure. The load was introduced through a single hydraulic actuator in conjunction with a steel spreader beam, positioned to apply symmetric loads 1025 mm from each support. The specimen’s geometry, internal reinforcement and prestressing layout, as well as the loading arrangement, are schematically detailed in Figure 12.

Owing to the geometric, material, and boundary condition symmetry inherent in the prestressed I-beam specimens tested by Rabczuk and Eibl [73], the finite element simulation was confined to a quarter-model domain. Symmetric boundary conditions were enforced along two orthogonal planes to accurately replicate the behavior of the full system while optimizing computational efficiency. The concrete domain was discretized using eight-node linear brick elements with reduced integration (C3D8R), whereas the internal reinforcement and prestressing tendons were idealized as axial load-carrying members using two-node truss elements (T3D2).

**Figure 13:**
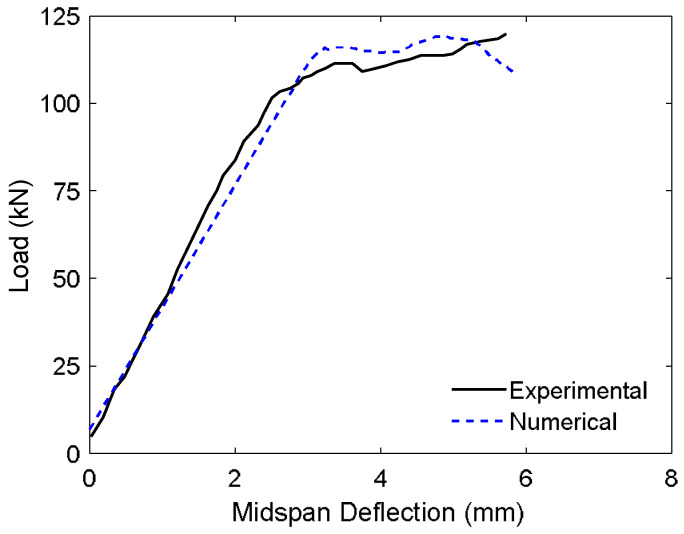
Experimental [73] and numerical results for a prestressed concrete beam.

Figure 13 shows the load–deflection response obtained from the finite element simulation alongside the corresponding experimental results for the prestressed concrete I-beam. The numerical model exhibits a high degree of agreement with the experimental data, accurately capturing both the global structural behavior and the ultimate load capacity. The FE analysis predicted an ultimate load of 119.17 kN, closely matching the experimentally measured value of 119.76 kN, with a minimal discrepancy of just 0.49%. Similarly, the maximum displacement was predicted to be 5.85 mm compared to the experimental value of 5.72 mm, yielding only a 2.30% difference.

Although a slight deviation was observed in the form of an earlier reduction in load-bearing capacity and marginal overestimation of displacement, these differences can be attributed to idealizations in the material models and the assumption of perfect bond between steel reinforcement and concrete. Nonetheless, the model successfully reproduced the key response characteristics, thus confirming its reliability in simulating prestressed flexural systems. This strong correlation underscores the robustness and predictive accuracy of the adopted finite element approach in representing the nonlinear behavior of prestressed concrete under flexural loading.

#### 5.1.3. CFRP-Retrofitted Prestressed Concrete Slab

**Figure 14:**
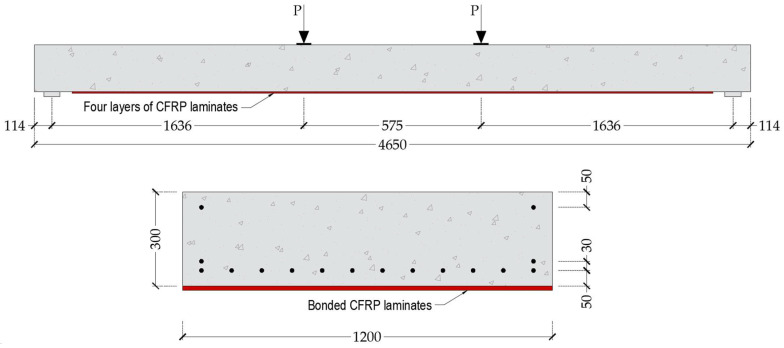
Geometry of the CFRP-strengthened prestressed concrete slab tested by Shahawy et al. [74] (dimensions in mm).

Shahawy et al. [74] conducted a comprehensive investigation into the use of CFRP materials for strengthening prestressed concrete elements, with particular emphasis on slab-type bridge systems. Their experimental program involved a full-scale pretensioned solid slab bridge that was retrofitted by externally bonding CFRP laminates along the tension face. The resulting test data served as a benchmark for validating the fidelity of the finite element model developed in the present study with the aim of simulating the structural response of CFRP-strengthened prestressed members. The geometric configuration of the retrofitted slab and the corresponding experimental setup are illustrated in Figure 14.

The bridge slab examined in the test had overall dimensions of 1200 mm in width, 300 mm in depth, and 4650 mm in span length. At the time of testing, the concrete used in casting the specimen exhibited a compressive strength of 41.37 MPa. Prestressing was introduced through 16 low-relaxation steel strands, each 13 mm in diameter, which were tensioned to an initial force of 138 kN per strand. These strands were characterized by a specified ultimate tensile strength of 1862 MPa and an assumed elastic modulus of 190 GPa, consistent with standard properties for high-strength prestressing steel.

The experimental program employed a four-point bending configuration over a simply supported span, with loading applied symmetrically at a distance of 1750 mm from each support using dual hydraulic actuators. Following the initial loading phase, during which the solid slab was intentionally pre-cracked, the specimen was retrofitted with four unidirectional CFRP laminate layers. Each laminate measured 0.17 mm in thickness, with fibers aligned along the longitudinal axis of the slab to optimize flexural reinforcement. The mechanical properties of the CFRP laminates were defined by an average tensile strength $\left(\bar{X}\right)$ of 2758 MPa and a longitudinal elastic modulus $\left(E_{11}\right)$ of 141.34 GPa. Additional orthotropic material parameters were assumed as follows: $\bar{Y}$ = $\bar{S}$ = 27.58 MPa, $\bar{X^{\prime }}$ = $\bar{Y^{\prime }}$ = −27.58 MPa, $E_{22}$ = $G_{12}$ = 1413 MPa, and Poisson’s ratio ν = 0.21.

Consistent with previously validated modeling strategies, the FE analysis was conducted using a quarter-symmetry model in which the symmetry boundary conditions were imposed along two orthogonal planes to reduce computational demand while preserving geometric and mechanical fidelity. The prestressed concrete slab was discretized using eight-node brick elements with reduced integration (C3D8R), and the internal prestressing strands were idealized as axial truss elements (T3D2). The CFRP laminates were represented using four-node shell elements (S4R), with four distinct layers incorporated into the model. A perfect bond was assumed, both between the CFRP layers themselves and between the laminates and the concrete substrate, thereby eliminating interfacial slip.

**Figure 15:**
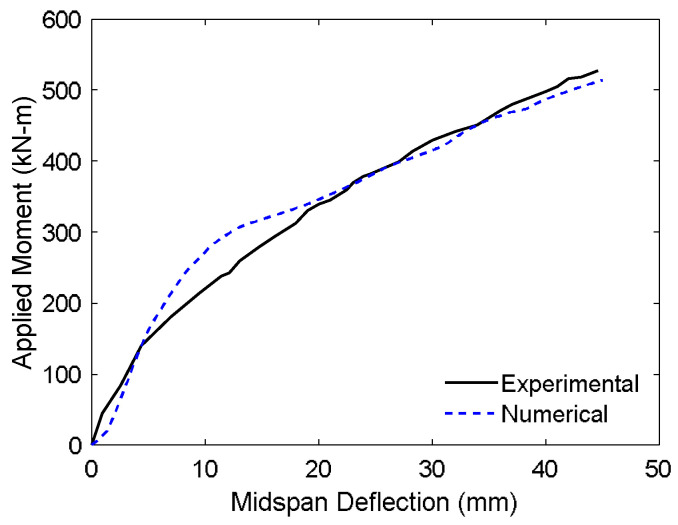
Comparison of numerical and experimental [74] moment–deflection behavior of the prestressed slab specimen.

The simulated flexural response of the strengthened slab is illustrated in Figure 15, which presents the moment–deflection behavior at the loading point. The FE model showed strong agreement with experimental observations, accurately capturing both the global response and ultimate capacity. The simulated ultimate bending moment of 513.65 kN·m was very close to the experimentally measured value of 527.59 kN·m, with a deviation of only 2.54%. Similarly, the maximum displacement was predicted as 45.00 mm compared to 44.60 mm in the experiment, corresponding to a minimal difference of 0.89%.

These small discrepancies can be attributed to modeling simplifications, such as the assumption of perfect bond and idealized material behavior, which may slightly influence stiffness degradation and post-peak softening. Nonetheless, the close agreement affirms the robustness of the proposed modeling framework in replicating the flexural behavior of CFRP-strengthened prestressed concrete systems.

### 5.2. Parametric Study of CFRP-Strengthened Prestressed Concrete T-Beams

The validity of the FE model developed in this study was established through comparison with the experimental benchmarks detailed in Section 5.1. Following successful validation, the model was employed to investigate the influence of CFRP strengthening on the flexural performance of prestressed T-beams subjected to three loading scenarios: reversed cyclic loading, and near-fault and far-field ground motions. While cyclic behavior was simulated using prescribed displacement cycles, the near-fault and far-field seismic actions were introduced through displacement time–history inputs derived from recorded ground motions.

#### 5.2.1. Response Under Reversed Cyclic Loading

##### Hysteresis Responses

**Figure 16:**
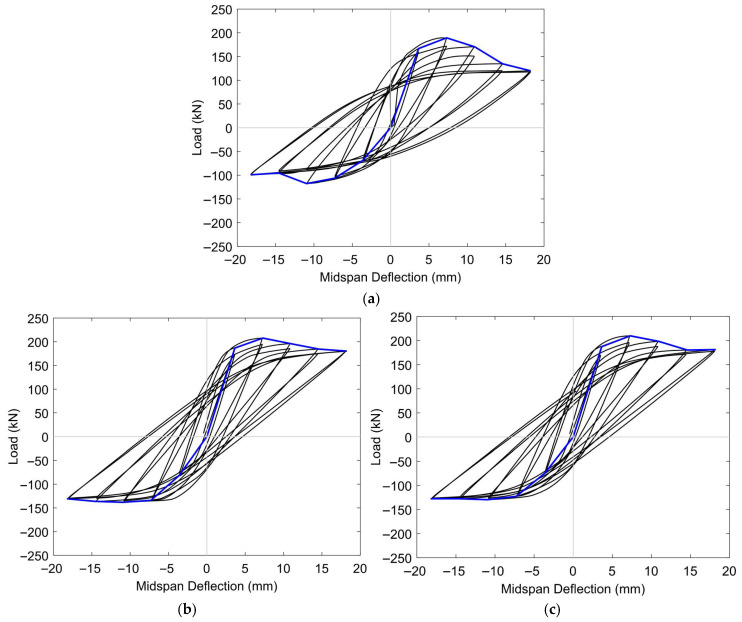
Responses of specimens under a low level of initial prestressing, with black lines representing hysteresis curves and blue lines indicating the envelope curves: (**a**) BN-L-C; (**b**) BH-L-C; (**c**) BF-L-C.

**Figure 17:**
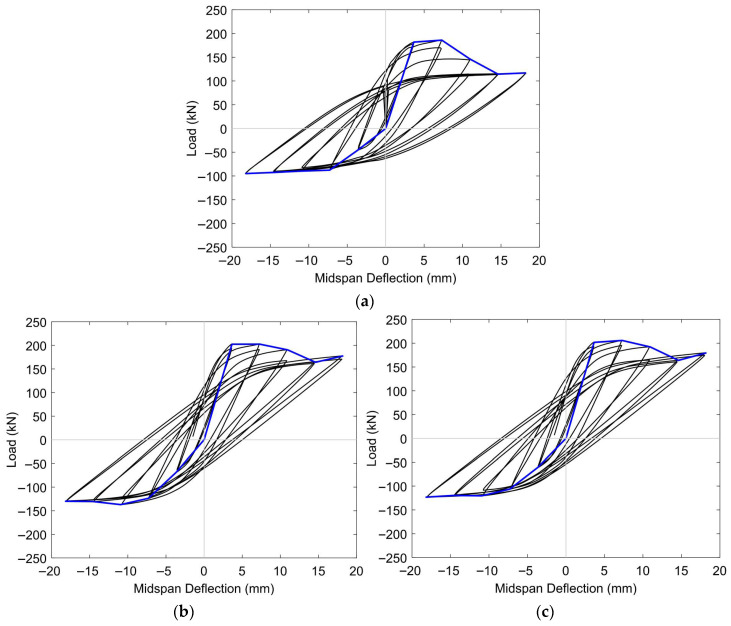
Responses of specimens under a high level of initial prestressing, with black lines representing hysteresis curves and blue lines indicating the envelope curves: (**a**) BN-H-C; (**b**) BH-H-C; (**c**) BF-H-C.

Figure 16 and Figure 17 illustrate the hysteresis responses of prestressed concrete T-beams subjected to reversed cyclic loading under two levels of initial prestressing (low and high). Each set included an unstrengthened control specimen (BN-L-C and BN-H-C), a beam retrofitted with half-embedded CFRP rods (BH-L-C and BH-H-C), and another with fully embedded CFRP rods (BF-L-C and BF-H-C). These curves provide insight into the cyclic behavior of each configuration, revealing differences in ductility, energy dissipation, and stiffness degradation with changes in the level of prestressing and the embedment strategy.

Under a low level of initial prestressing, the control specimen BN-L-C underwent narrow, pinched hysteresis loops with increasing asymmetry and residual deformation as the drift increased, reflecting limited energy dissipation and early stiffness degradation. The BH-L-C specimen showed moderate improvements, with slightly wider and more stable loops, although minor pinching remained due to bond degradation. In contrast, the BF-L-C beam exhibited the most stable cyclic response, characterized by wide, symmetric loops and minimal stiffness loss, highlighting the benefit of full CFRP embedment in regard to enhancing ductility and load redistribution under low prestress conditions.

Under a high level of initial prestressing, all specimens exhibited increased stiffness and load capacity in the early stages of loading. However, the BN-H-C specimen still suffered from noticeable pinching and a reduction in loop area at large displacements, indicating that prestressing alone does not provide sufficient energy dissipation. The BH-H-C beam, in contrast, showed significant performance gains, including improved symmetry and reduced degradation. The BF-H-C specimen again outperformed the others, maintaining large, stable loops with high energy dissipation throughout the loading cycles; this finding emphasizes the superior bond efficiency of full-depth CFRP embedment under high prestressing forces.

##### Envelope Curves

**Figure 18:**
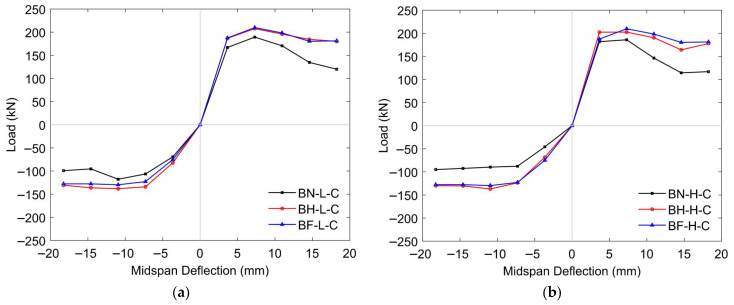
Envelope curves for specimens subjected to reversed cyclic loading under varying initial prestressing levels: (**a**) low initial prestressing level; (**b**) high initial prestressing level.

**Figure 19:**
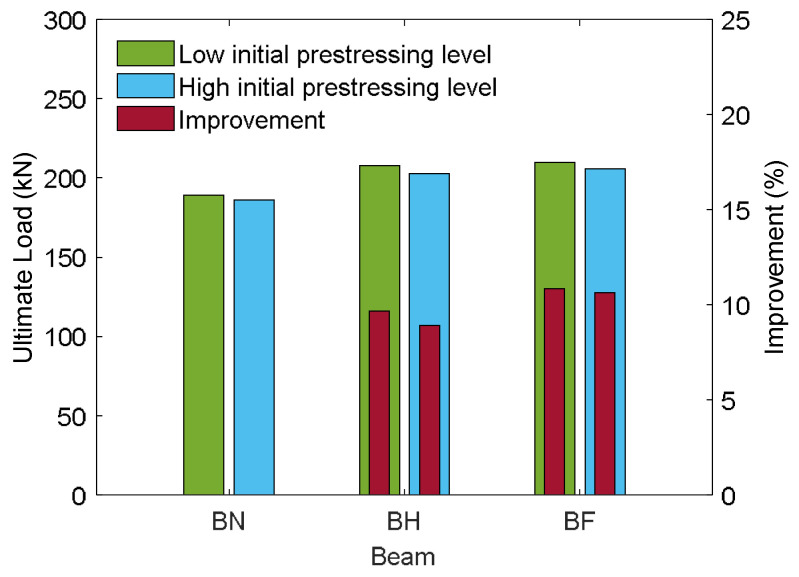
Ultimate loads and improvements in CFRP strengthening for specimens under reversed cyclic loading at low and high levels of initial prestressing.

The envelope curves in Figure 18 provide valuable insights into the global flexural response of prestressed concrete T-beams under reversed cyclic loading and highlight the influence of both the initial prestressing levels and CFRP embedment depths. By plotting only the peak lateral force values at the maximum deflection for each cycle, these curves simplify while effectively capturing the essential aspects of the load–deflection behavior, such as strength progression, degradation trends, and residual capacity. To further clarify and quantify the relative strength gains across specimens, Figure 19 shows a comparative bar chart that summarizes the ultimate loads and improvement percentages under both levels of prestressing.

Under low initial prestressing, the control specimen (BN-L-C) exhibited the lowest peak strength at 189.34 kN, with a gradual rise followed by strength degradation. Strengthening led to noticeable improvements: BH-L-C reached 207.68 kN (9.69% increase), while BF-L-C achieved the highest capacity at 209.87 kN (10.84% increase). These gains are primarily attributed to the tensile contribution of the CFRP rods, which significantly enhanced the beam’s flexural resistance [29,41,42,43,44]. The strengthened beams also demonstrated improved inelastic behavior, enabling greater energy dissipation and a more controlled distribution of seismic demand [79]. The envelope curves for BH-L-C and BF-L-C illustrate these effects, showing broader, more stable loops that indicate enhanced ductility and reduced post-peak strength degradation.

At the high initial prestressing level, the unstrengthened specimen (BN-H-C) reached an ultimate load of 185.98 kN, slightly lower (−1.77%) than its low-prestress counterpart. Both strengthened specimens still benefited from CFRP reinforcement, with BH-H-C achieving 202.54 kN (an 8.90% gain over BN-H-C) and BF-H-C attaining 205.74 kN (a 10.62% gain). However, compared to their low-prestress counterparts, the strengthened beams showed slight reductions in peak capacity (−2.47% for BH-H-C and −1.97% for BF-H-C). These results suggest that CFRP strengthening becomes less efficient under higher prestress levels. While higher prestress improves initial stiffness, it reduces deformability, restricts crack opening, and limits the full mobilization of CFRP reinforcement. Consequently, the contribution of CFRP to strength enhancement is diminished, and earlier stiffness degradation further reduces its effectiveness. This interaction highlights the sensitivity of CFRP efficiency to prestress level and the need to carefully consider prestressing conditions in the design of strengthening strategies.

##### Stiffness Degradation

**Figure 20:**
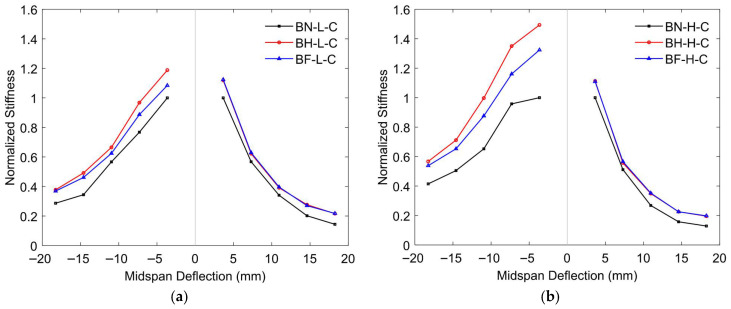
Stiffness degradation of specimens under reversed cyclic loading at varying levels of initial prestressing: (**a**) Low initial prestressing level; (**b**) High initial prestressing level.

The stiffness degradation behavior of prestressed concrete T-beams subjected to reversed cyclic loading is shown in Figure 20 and reveals the influence of both CFRP strengthening and initial prestressing levels on structural resilience. Stiffness values were normalized to the peak response and plotted against midspan deflection to illustrate the rate and extent of degradation as cyclic loading progressed.

In the case of low initial prestressing [Figure 20a], the control beam (BN-L-C) exhibited the most rapid stiffness degradation, reflecting limited energy dissipation and progressive deterioration of the material. Following CFRP strengthening, the half-embedded beam (BH-L-C) and fully embedded beam (BF-L-C) achieved higher stiffness throughout the loading cycles, particularly in the negative deflection range. BH-L-C again reached the highest normalized stiffness in the early cycles, suggesting effective early-stage load transfer, while BF-L-C demonstrated more stable stiffness retention over a wider deflection range. This indicates that both half and full embedment contribute to stiffness preservation, though the benefits of full embedment become more evident under larger deformations.

For beams with high initial prestressing [Figure 20b], similar trends were observed but with slightly higher initial stiffness across all specimens due to the prestress-induced axial force. The control specimen (BN-H-C) still showed the most pronounced reduction in stiffness. In contrast, the strengthened beams (BH-H-C and BF-H-C) exhibited higher stiffness retention [79,80,81,82], with BH-H-C again showing the highest normalized stiffness under early deflections. However, the gap between BH-C-H and BF-C-H narrowed as the deflection increased, indicating that the prestress level complements the CFRP reinforcement in maintaining stiffness under larger cyclic demands.

##### Displacement Ductility

In assessing the seismic response of prestressed concrete structures, ductility serves as a fundamental indicator of the member’s capacity to undergo plastic deformation beyond its elastic limit. It is commonly defined as the ratio of ultimate to yield displacement. In this study, the global ductility (*μ*) of each specimen was evaluated by accounting for both positive and negative displacement cycles associated with their corresponding ultimate loads. The cumulative ductility in the two loading directions is formulated in Equation (12).

(12) $$\mu =\frac{\left|\Delta_{u}^{+}\right|}{\left|\Delta_{y}^{+}\right|}+\frac{\left|\Delta_{u}^{-}\right|}{\left|\Delta_{y}^{-}\right|}$$

where $\Delta_{u}^{+}\;$ and $\Delta_{u}^{-}\;$ denote the ultimate displacements in the positive and negative directions, while $\Delta_{y}^{+}$ and $\Delta_{y}^{-}$ correspond to the respective yield displacements. Both yield and ultimate points were identified using the equivalent elastoplastic system approach. The yield displacement ( $\Delta_{y}$ ) was obtained by locating the intersection between a secant line from the origin to 75% of the peak load and a horizontal line at the peak load level. In contrast, the ultimate displacement ( $\Delta_{u}$ ) was defined as the terminal point on the load–displacement curve. Table 6 summarizes the displacement ductility (*μ*) of the evaluated specimens under both low and high levels of initial prestressing.

Across all cases, ductility values ranged narrowly between 6.90 and 7.30, reflecting a stable and consistent deformation capacity of the prestressed beams, irrespective of prestress level or CFRP configuration. Under low prestress, the control beam BN-L-C exhibited a ductility of 6.90, while strengthened beams BH-L-C and BF-L-C both reached 7.10. This modest increase demonstrates that CFRP reinforcement contributed to maintaining ductile response without compromising deformation capacity, despite enhancing strength. The nearly identical ductility values of BH-L-C and BF-L-C further suggest that half and full embedment provided comparable benefits in terms of deformation demand under low prestress.

When the prestressing level was increased, ductility values rose slightly. The unstrengthened specimen BN-H-C achieved 7.20, indicating that higher prestress improved deformation capacity in the control condition. Strengthened beams BH-H-C and BF-H-C reached 7.30 and 7.10, respectively, showing only marginal differences compared with their low-prestress counterparts. These results indicate that while higher prestress enhances ductility by delaying crack propagation and improving energy absorption, the incremental benefit of CFRP reinforcement becomes less pronounced at high prestress. Taken together, the findings confirm that CFRP strengthening does not reduce ductility and, in some cases, provides slight improvements.

##### Damage Contours

**Figure 21:**
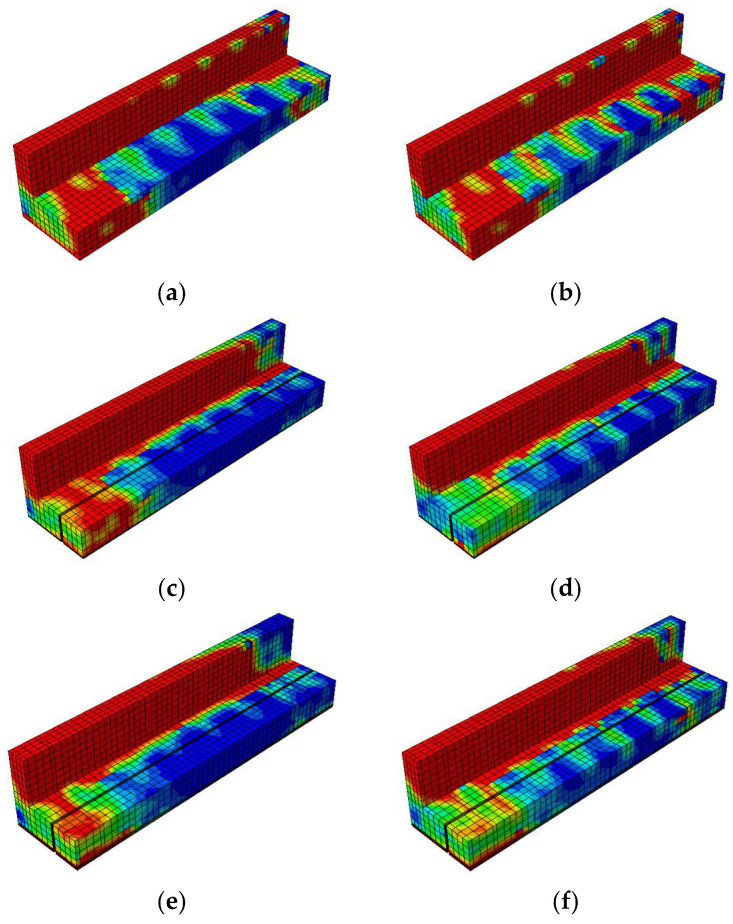
Comparison of compressive damage contours. Color gradients represent the severity of compressive damage, with red indicating significant damage and blue indicating minimal or no damage. Black lines show the FE mesh used for structural discretization. Specimens shown: (**a**) BN-L-C; (**b**) BN-H-C; (**c**) BH-L-C; (**d**) BH-H-C; (**e**) BF-L-C; (**f**) BF-H-C.

**Figure 22:**
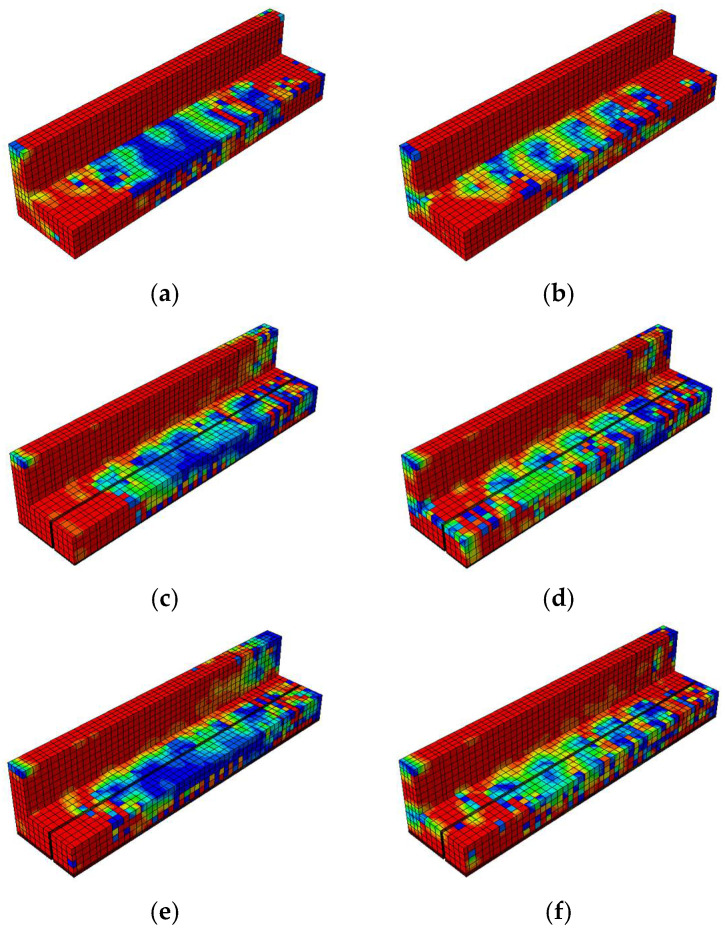
Comparison of tensile damage contours. Color gradients reflect the extent of tensile damage, with red areas indicating severe cracking and blue areas representing undamaged regions. The black mesh lines illustrate the FE discretization of the structural model. Specimens shown: (**a**) BN-L-C; (**b**) BN-H-C; (**c**) BH-L-C; (**d**) BH-H-C; (**e**) BF-L-C; (**f**) BF-H-C.

The damage contour distributions in Figure 21 and Figure 22 provide important insights into how variations in prestressing levels and CFRP strengthening affect the behavior of prestressed concrete T-beams under cyclic loading. The compressive and tensile damage patterns are represented using the CDP model parameters $d_{c}$ and $d_{t}$ , respectively. The color gradients indicate the severity of damage, where red corresponds to values approaching one (severe crushing or cracking) and blue denotes values near zero (minimal or no damage). The overlaid black lines represent the finite element mesh used for discretization. A horizontal comparison (left to right) reveals the influence of the initial prestressing level, whereas a vertical comparison (top to bottom) highlights the effectiveness of the CFRP strengthening configurations.

The compressive damage shown in Figure 21 indicates that the unstrengthened specimen with low initial prestress (BN-L-C) underwent severe damage concentrated at the web and loading point, characterized by extensive red zones. Increasing the prestress level to 70% (BN-H-C) helped to reduce the compressive damage in the lower flange but had a limited effect on the upper web. A horizontal comparison shows that a greater level of prestress redistributed damage away from the web and alleviated stress concentrations at the flange. In the vertical direction, CFRP strengthening significantly reduced compressive damage, with the CFRP sheets promoting more uniform stress distribution along the web. The BF specimens (BF-L-C and BF-H-C) showed the least damage overall, a finding that can be attributed to the combined effect of CFRP confinement on the web and rods at the flange. This mitigation was slightly more pronounced at the higher prestressing level, suggesting a synergistic interaction between prestress and CFRP confinement in managing compressive damage.

The tensile damage shown in Figure 22 indicates that the control specimens again underwent widespread cracking along the bottom fiber and web under cyclic flexural tension. The use of CFRP rods and sheets significantly mitigated this damage, especially in the fully embedded configurations. BF-L-C and BF-H-C exhibited more uniform and less intense damage patterns, with red zones confined to limited areas. A vertical comparison highlights that CFRP sheets helped to absorb tensile stress at the web, while CFRP rods enhanced the tensile resistance at the flange. Compared to BH specimens, in which half embedment was used, the BF beams demonstrated clearer advantages in terms of reducing crack intensity and propagation. A horizontal comparison shows that higher prestressing further reduced tensile damage, particularly at the flange, although its effect on the web was less evident.

#### 5.2.2. Response Under Near-Fault Ground Motion

**Figure 23:**
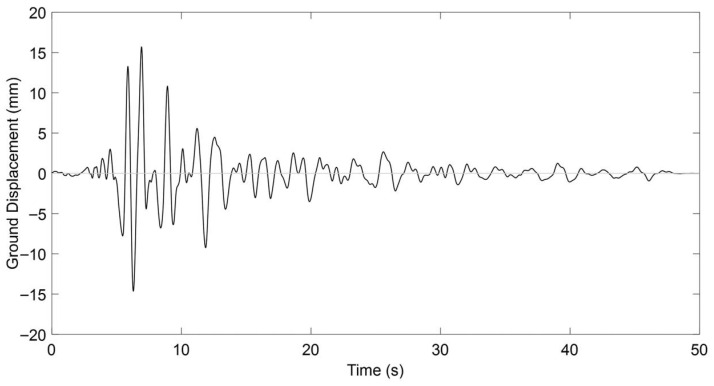
Near-fault ground displacement at Taichung, Taiwan.

A critical metric for delineating the near-fault region involves evaluating the ratio between the fault rupture dimension and the distance from the seismic source to the observation site [83]. According to Bary and Rodriquez-Marek [84], this proximity zone typically extends no more than 20 km from the fault rupture, beyond which the characteristic near-fault effects begin to attenuate significantly. This spatial limitation arises from the natural decay of near-fault phenomena with increasing distance, compounded by factors such as earthquake magnitude and localized geotechnical conditions [85]. In the present study, the near-fault seismic input was derived from the Chi-Chi earthquake record obtained in Taichung, Taiwan, as shown in Figure 23.

The record was scaled on the basis of peak ground acceleration (PGA) to ensure compatibility with the target seismic intensity level adopted in this study. This approach allowed the displacement demands to be consistent with the expected response range of prestressed T-beams of the studied size and reinforcement details. Following scaling, the near-fault ground motion produced a maximum displacement of 15.71 mm, which corresponds to a realistic demand level for members of this scale and reinforcement ratio. While no spectral matching was applied, the PGA-based scaling method ensured that the overall energy content and displacement ductility demands remained within the range typically observed for near-fault events acting on medium-span concrete beams, thereby maintaining both computational practicality and engineering relevance.

##### Load Characteristics

**Figure 24:**
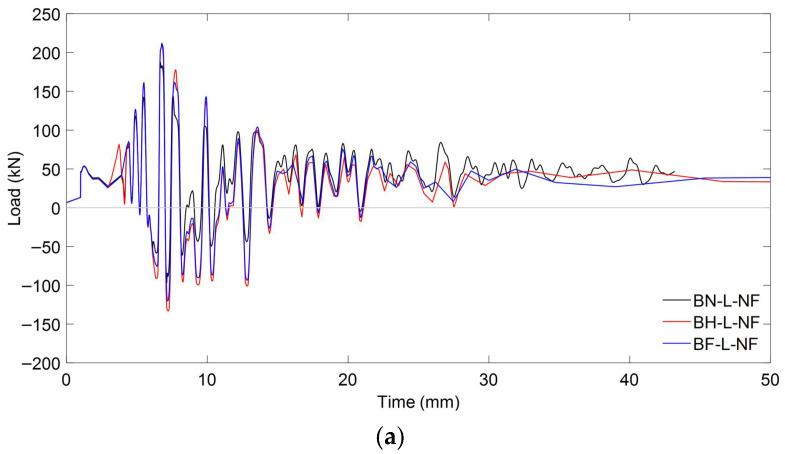

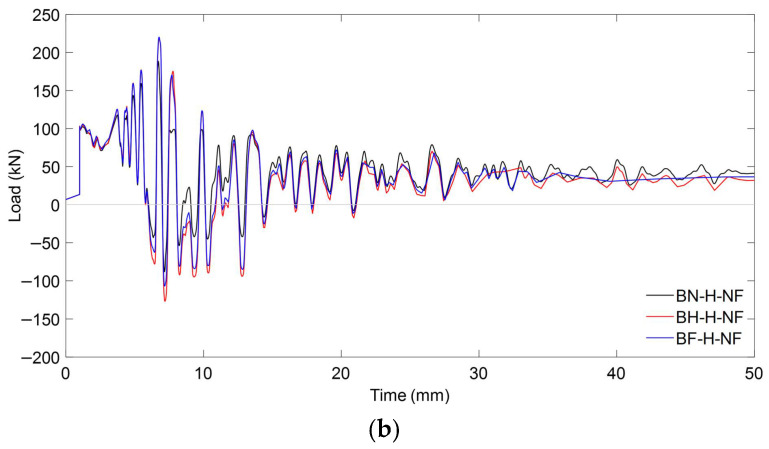
Load characteristics of specimens under near-fault ground motion at varying initial prestressing levels: (**a**) Low initial prestressing level; (**b**) High initial prestressing level.

Figure 24a illustrates the time–history load responses of specimens subjected to near-fault ground motion under low initial prestressing levels. A general trend is observed in which all specimens experience significant load fluctuations in the early seconds of excitation, characteristic of near-fault pulse-like motions. The unstrengthened specimen (BN-L-NF) shows lower peak amplitudes and dissipates energy more quickly, whereas the strengthened specimens, BH-L-NF and BF-L-NF, maintain higher and more sustained load responses. This indicates that CFRP strengthening, even under low prestressing conditions, enhances energy absorption and prolongs structural engagement during the strong-motion phase of near-fault events.

With high initial prestressing, as shown in Figure 24b, the seismic response becomes more stable and intensified. All beams show slightly faster damping of oscillations, and the amplitude of the early load peaks increases compared to their low prestress counterparts. The CFRP-strengthened beams (BH-H-NF and BF-H-NF) show clearer load recovery after peak cycles, reflecting improved post-yield resilience. The addition of prestressing appears to contribute to a stiffer initial response, while the presence of CFRP helps to mitigate stiffness degradation and control dynamic displacement, particularly when full-depth embedment is applied.

**Figure 25:**
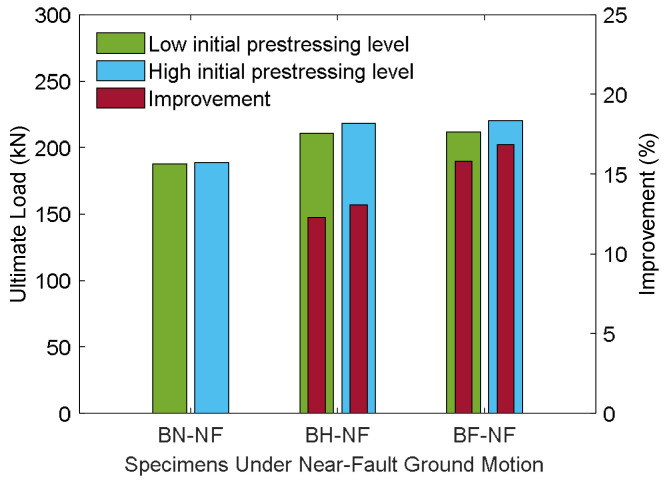
Ultimate loads and improvements due to CFRP strengthening for specimens under near-fault ground motion at low and high levels of initial prestressing.

Figure 25 provides quantitative confirmation of the flexural performance under near-fault excitation, highlighting the distinct roles of prestress level and CFRP strengthening. For specimens with low initial prestressing, the ultimate load increased from 187.40 kN (BN-L-NF) to 210.42 kN (BH-L-NF) and 211.90 kN (BF-L-NF), corresponding to improvements of 12.29% and 13.07%, respectively. These results clearly indicate that CFRP strengthening, even with half embedment depth, substantially enhances structural resistance under severe cyclic demands characteristic of near-fault ground motions. In contrast, the control beam exhibited only a marginal increase in peak load when prestress was raised, reaching 188.48 kN. This suggests that prestressing alone provides limited benefit under near-fault conditions, where rapid load reversals and high inelastic demands dominate the structural response.

When CFRP was introduced, however, the effect of prestress became more evident. The strengthened BH-H-NF and BF-H-NF beams achieved ultimate loads of 218.22 kN and 220.22 kN, corresponding to gains of 15.78% and 16.84%, respectively, over the high-prestress control. The superior performance of the BF configuration can be attributed to the improved stress transfer and anchorage offered by full embedment, which delays debonding and ensures better utilization of CFRP capacity. Taken together, these findings underscore that CFRP strengthening plays the dominant role in enhancing structural resistance under near-fault loading, while prestress acts as a secondary contributor whose benefits are best realized when paired with sufficient CFRP embedment. This interaction highlights the importance of optimizing both parameters to achieve resilient retrofit strategies for prestressed concrete members in regions susceptible to near-fault seismic hazards.

##### Damage Contours

**Figure 26:**
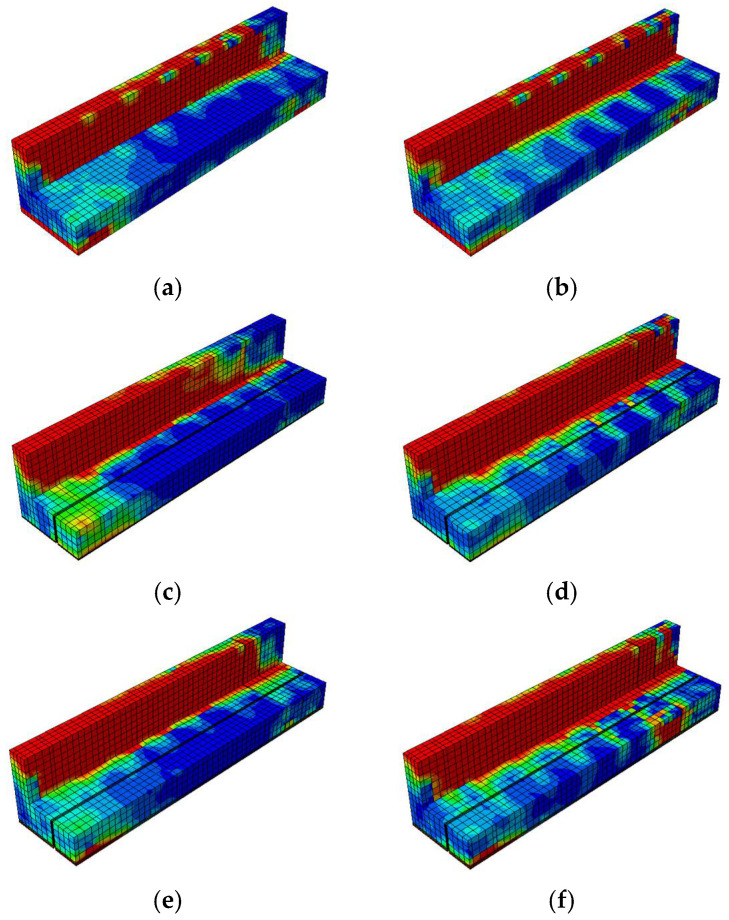
Comparison of compressive damage contours. Color gradients represent the severity of compressive damage, with red indicating significant damage and blue indicating minimal or no damage. Black lines show the FE mesh used for structural discretization. Specimens shown: (**a**) BN-L-NF; (**b**) BN-H-NF; (**c**) BH-L-NF; (**d**) BH-H-NF; (**e**) BF-L-NF; (**f**) BF-H-NF.

**Figure 27:**
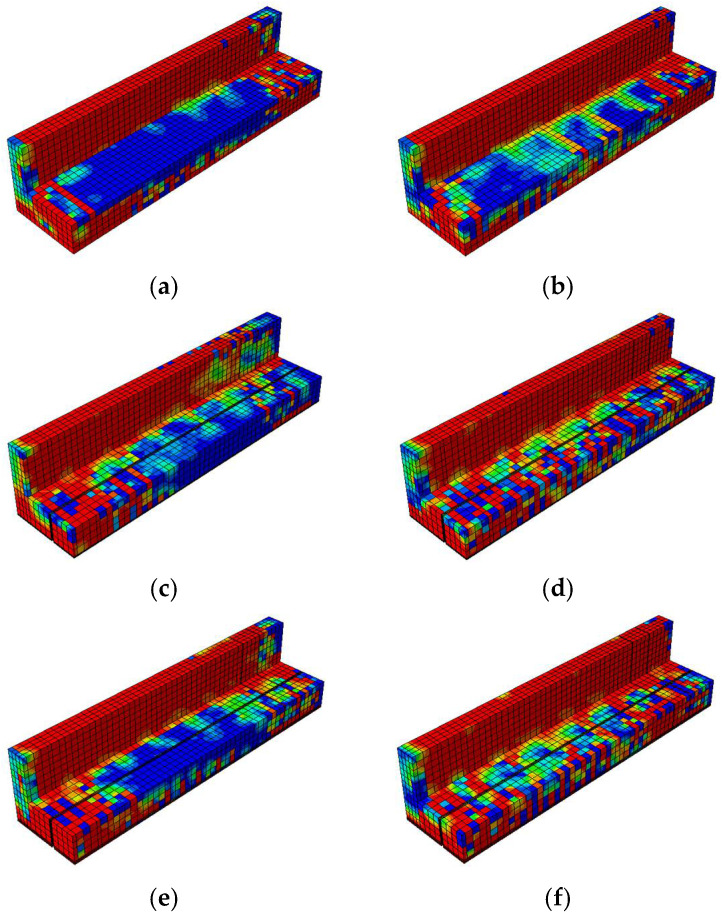
Comparison of tensile damage contours. Color gradients reflect the extent of tensile damage, with red areas indicating severe cracking and blue areas representing undamaged regions. The black mesh lines illustrate the FE discretization of the structural model. Specimens shown: (**a**) BN-L-NF; (**b**) BN-H-NF; (**c**) BH-L-NF; (**d**) BH-H-NF; (**e**) BF-L-NF; (**f**) BF-H-NF.

Figure 26 and Figure 27 enable a comparative visualization of the compressive and tensile damage patterns in prestressed concrete T-beams subjected to near-fault ground motion, highlighting the combined effects of CFRP strengthening and varying initial prestressing levels. In terms of compressive damage (Figure 25), the control specimens (BN-NF) under low prestress exhibited severe damage concentrated at the web and top flange, which was only moderately alleviated under high prestress conditions.

This shift in the damage pattern reflected the redistributive effect of prestressing, although large red zones still remained in critical regions. In contrast, strengthened specimens using CFRP rods (BH-NF) showed more localized stress control, particularly around the flange, with improvements becoming more visible at high prestress levels. The most significant mitigation occurred in the BF-NF specimens, where the combination of full-depth CFRP embedment and high prestress resulted in the most uniform and least severe compressive damage distribution. These observations affirm that CFRP sheets provide effective confinement, and when combined with sufficient prestress, enhance the beam’s ability to resist compressive failure under seismic excitation.

Similar trends were observed for the tensile behavior (Figure 27). The control beams exhibited extensive cracking at the lower fiber and web in both prestress scenarios, with only a minor reduction at a high level of prestress. These red zones, which are particularly concentrated along the tension face and near the web, suggest the inability of unstrengthened sections to adequately resist seismic-induced flexural demands. Strengthened BH beams exhibited improved tensile resistance due to the partial engagement of CFRP rods, although stress concentration remained evident, particularly on the web.

Full-depth CFRP embedment in BF specimens significantly minimized tensile damage across both prestress levels, with BF-H-NF showing the most uniform distribution and least crack severity. These results indicate that CFRP rods not only reinforce the tensile zone but also limit crack propagation more effectively when fully embedded. When taken together, the damage contour comparisons indicate that while increased prestressing contributes to damage redistribution, the greatest enhancements in structural resilience are achieved through synergy between an optimized level of prestress and complete CFRP strengthening.

#### 5.2.3. Response Under Far-Field Ground Motion

**Figure 28:**
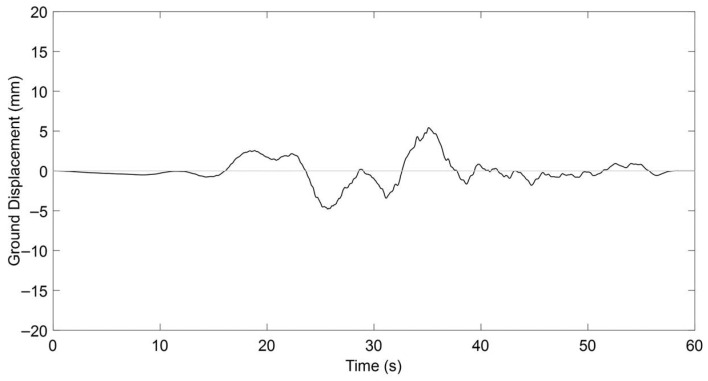
Far-field ground displacement recorded in Yilan, Taiwan.

Far-field ground motions are typically characterized by sustained energy release and broader frequency content compared to their near-fault counterparts. Whereas near-fault earthquakes are often dominated by intense velocity pulses and concentrated low-frequency energy, far-field events tend to produce higher-frequency content over an extended duration [86,87]. Despite their lower peak accelerations, near-fault motions, particularly those with pulse-like signatures, are known to induce more severe structural responses due to their abrupt energy input. For the far-field excitation, seismic input was taken from the Chi-Chi earthquake record at Yilan, Taiwan, as shown in Figure 28.

Prior to use in the analysis, the record was normalized according to its PGA so that the input motion would be consistent with the design-level seismic demand assumed for the studied beam size and reinforcement configuration. No spectral matching was applied; instead, PGA-based scaling was chosen to preserve the natural frequency content and energy distribution of the original record. The resulting displacement history yielded a maximum amplitude of 5.43 mm, which falls within the range of realistic demand for medium-span prestressed T-beams under far-field shaking. Although this scaling approach simplifies the representation of seismic demand, it ensures a reasonable balance between computational efficiency and the faithful reproduction of structural response under far-field ground motions.

##### Load Characteristics

**Figure 29:**
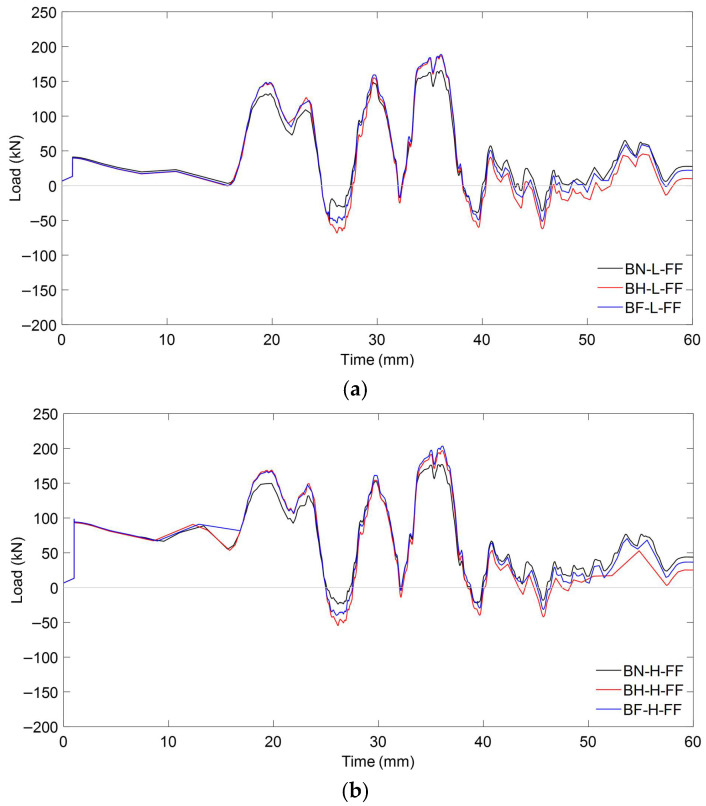
Load characteristics of specimens under far-field ground motion at varying initial prestressing levels: (**a**) Low initial prestressing level; (**b**) High initial prestressing level.

Based on the dynamic response profiles illustrated in Figure 29a, the load characteristics of specimens subjected to far-field ground motion under low initial prestressing levels demonstrate distinguishable oscillation patterns. The unstrengthened beam (BN-L-FF) displayed irregular amplitude variations with early reductions in load peaks, highlighting limited energy dissipation and structural stiffness. In contrast, the strengthened specimens, particularly BF-L-FF, exhibited more stable responses with delayed amplitude attenuation, suggesting enhanced resilience. The presence of CFRP reinforcement, especially with full-depth embedment, contributed to prolonged stiffness retention and improved control over cyclic degradation effects in far-field conditions.

When evaluating specimens with high initial prestressing levels, as presented in Figure 29b, a generally stiffer response is observed across all configurations. The prestressed beams exhibited smaller peak-to-peak variations and faster damping rates compared to their low-prestress counterparts, indicating improved stability and energy absorption. Of these, BF-H-FF maintained the most consistent reduction in amplitude, a finding that underscores the effectiveness of combining high prestress with full-depth CFRP rods. The comparison confirms that a combination of prestress and full-depth CFRP enhancement provides superior load modulation and better control over post-peak behavior under the prolonged seismic excitation typical of far-field scenarios.

**Figure 30:**
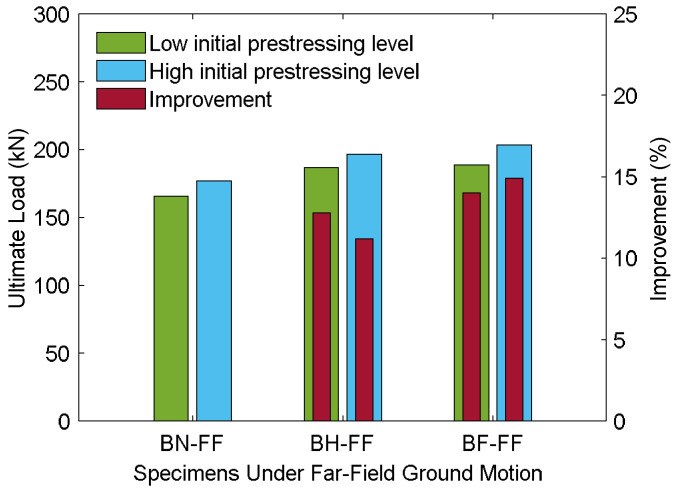
Ultimate loads and improvements in CFRP strengthening for specimens under far-field ground motion at low and high levels of initial prestressing.

Figure 30 provides a quantitative summary of flexural capacity under far-field excitation, illustrating the combined influence of prestress level and CFRP embedment. At low prestress, the unstrengthened BN-L-FF beam reached an ultimate load of 165.49 kN, while CFRP strengthening led to notable increases of 12.79% for BH-L-FF and 14.00% for BF-L-FF. These results confirm that CFRP reinforcement significantly enhances flexural resistance under far-field motions, with full embedment yielding the greatest improvement. When the prestress level was raised, capacity gains were also evident across all configurations: BN-H-FF increased by 6.91%, BH-H-FF by 5.37%, and BF-H-FF by 7.77% compared to their low-prestress counterparts. This indicates that prestress improves baseline strength and stiffness, enabling beams to better sustain seismic demands.

The BF configuration consistently provided the highest strength, achieving 203.32 kN and representing a combined improvement of 14.91%. This superior performance can be attributed to the enhanced bond area and stress distribution afforded by full embedment, which delays debonding and ensures more efficient CFRP utilization. Taken together, the findings highlight the synergy between higher prestress and full-depth CFRP embedment in maximizing flexural capacity under far-field loading. These insights reinforce the importance of embedment depth and prestress optimization as key design variables for practical retrofitting strategies aimed at improving the seismic resilience of prestressed concrete systems.

##### Damage Contours

**Figure 31:**
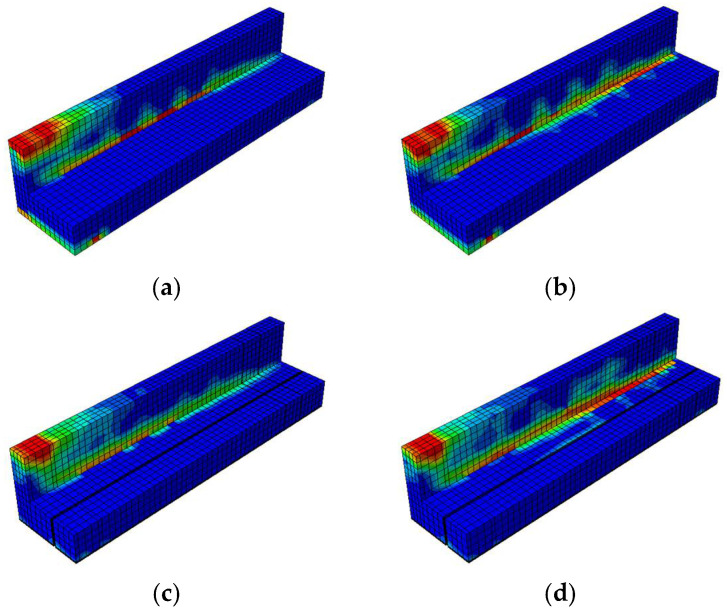

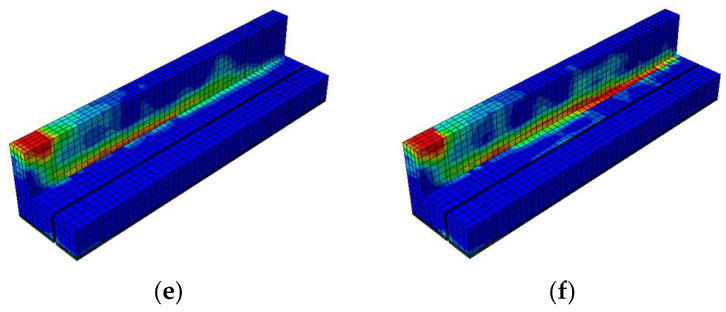
Comparison of compressive damage contours. Color gradients represent the severity of compressive damage, with red indicating significant damage and blue indicating minimal or no damage. Black lines show the finite element mesh used for structural discretization. Specimens shown: (**a**) BN-L-FF; (**b**) BN-H-FF; (**c**) BH-L-FF; (**d**) BH-H-FF; (**e**) BF-L-FF; (**f**) BF-H-FF.

A damage contour analysis offers a clear understanding of the effects of prestress levels and CFRP strengthening under far-field ground motion. As shown in Figure 31, compressive damage was most prominent in the control specimens (BN-L-FF and BN-H- FF), with red zones concentrated around the web and flange. An increase in the initial prestress from low to high (BN-L-FF to BN-H-FF) helped to reduce the spread of compressive damage, particularly at the web, although the damage near the flange remained significant.

The introduction of CFRP strengthening further enhanced structural performance. Both BH and BF specimens exhibited a notable decrease in compressive damage; this was especially true for the BF-H-FF specimen, where full-depth embedment and prestress combined to redistribute the stress more uniformly and to delay crushing. The consistent blue zones in these strengthened beams indicate effective confinement and stress mitigation under prolonged excitation.

**Figure 32:**
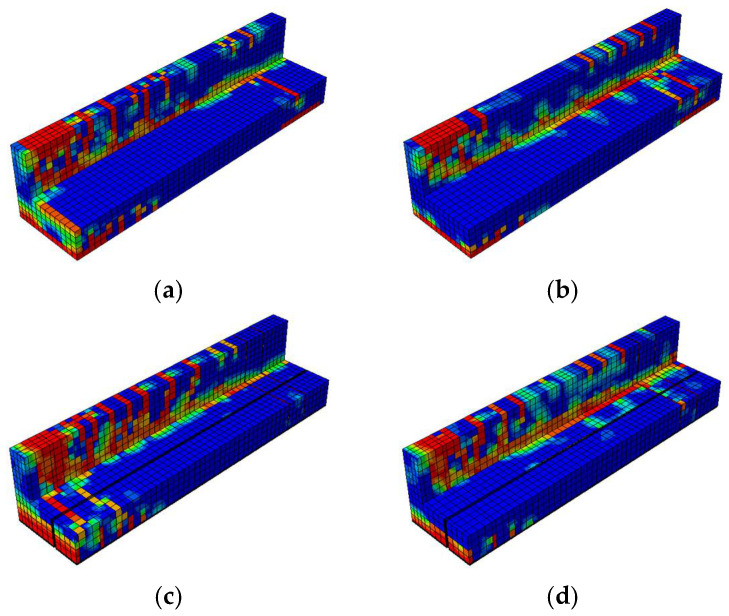

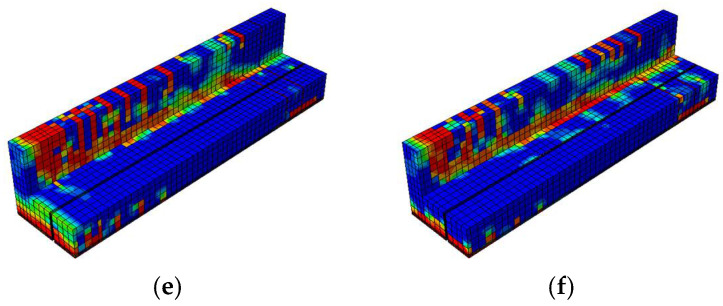
Comparison of tensile damage contours. Color gradients reflect the extent of tensile damage, with red areas indicating severe cracking and blue areas representing undamaged regions. The black mesh lines illustrate the finite element discretization of the structural model. Specimens shown: (**a**) BN-L-FF; (**b**) BN-H-FF; (**c**) BH-L-FF; (**d**) BH-H-FF; (**e**) BF-L-FF; (**f**) BF-H-FF.

Figure 32 shows similar trends in the tensile damage, where control specimens showed extensive cracking along the lower fiber and web, with limited relief from increased prestress alone. Half-embedded CFRP rods (BH specimens) provided moderate improvement by reducing the width and extent of cracking, while full-depth CFRP reinforcement (BF specimens) had significantly enhanced tensile resistance. The BF-H-FF configuration, in particular, displayed the most favorable distribution, with minimal red zones and well-confined damage, thus demonstrating the synergistic benefit of a high level of prestressing and comprehensive CFRP strengthening. These observations underscore the role of tailored retrofitting strategies in improving structural resilience under far-field seismic demand.

## 6. Conclusions

In this research, a finite element modeling framework was introduced that was designed to investigate the seismic response of prestressed concrete T-beams retrofitted in the negative moment region using NSM-CFRP rods and sheets. The numerical formulation incorporated the nonlinear constitutive behavior for concrete, reinforcing steel, and prestressing tendons, while CFRP materials were modeled with linear elasticity. The critical bonding mechanisms between CFRP and concrete were captured through the implementation of cohesive interaction laws, thereby enabling a realistic representation of the interfacial performance under load.

The performance of the model was rigorously validated through comparison with multiple experimental benchmarks. The calibration process encompassed RC T-beams subjected to reversed cyclic loading, as well as prestressed concrete I-beams and slabs strengthened with externally bonded CFRP systems. The close agreement between numerical predictions and observed experimental outcomes affirmed the robustness of the proposed model in regard to capturing the complex behavior of FRP-strengthened members under seismic demand.

With regard to the reversed cyclic response, CFRP strengthening was found to substantially improve the structural resilience, especially with full-depth embedment. At a low level of prestress, a 10.84% increase in ultimate load was achieved for BF-L-C, slightly higher than the 9.69% increase for BH-L-C. Although the highly prestressed control beam showed a minor reduction in strength, performance gains of 8.90% and 10.62% were maintained for the strengthened BH-H-C and BF-H-C specimens, respectively. Moreover, full embedment consistently led to better stiffness retention and reduced structural degradation, as reflected in the stiffness degradation plots and damage contour distributions.

Under near-fault ground motion conditions, CFRP strengthening remained highly effective, particularly when combined with optimized prestressing. Although the high-prestress control beam (BN-NF-H) exhibited only a marginal increase in peak load compared to its low-prestress counterpart, the inclusion of CFRP significantly enhanced performance, with ultimate loads of 218.22 kN and 220.22 kN for BH-NF-H and BF-NF-H, respectively, corresponding to strength gains of 15.78% and 16.84%, respectively, over BN-H-NF. Load time–history responses confirmed that strengthened specimens sustained higher amplitudes for longer durations, indicating enhanced energy absorption. Damage analysis also demonstrated that CFRP, especially in the BF configuration, helped to control cracking and distribute the stresses more evenly throughout the beam.

Under far-field ground motion, the observed trends similarly underscored the structural benefits of CFRP retrofitting. Under low prestress, strength increases of 12.79% and 14.00% were seen for BH-L-FF and BF-L-FF, respectively, compared to the control (165.49 kN). At a high level of prestressing, BF-H-FF achieved the highest load capacity of 203.32 kN, representing a total gain of 14.91%. In terms of response behavior, CFRP-strengthened beams exhibited improved amplitude stability and slower stiffness degradation, while the damage contour results indicated substantially mitigated compressive and tensile cracking, particularly for fully embedded systems. In terms of ductility, all specimens exhibited values within a narrow range of 6.90–7.30, confirming that CFRP strengthening did not compromise deformability; in some cases, slight enhancements were observed, particularly under low prestress. This indicates that the strengthened beams retained sufficient plastic deformation capacity, an essential parameter for seismic performance.

Altogether, the results demonstrated that NSM-CFRP strengthening significantly enhanced the seismic performance of prestressed concrete T-beams, even when only half embedment depth was feasible. These findings are particularly relevant for practical applications, as partial embedment is often the only viable option in existing structures where full-depth installation is constrained by cover thickness or construction limitations. The validated FE framework provides a reliable tool for assessing retrofit strategies under realistic seismic scenarios and supports the development of performance-based design guidelines. The outcomes of this study therefore contribute not only to advancing fundamental knowledge but also to informing engineers and practitioners about effective, resilient, and constructible solutions for extending the service life of aging prestressed concrete infrastructure in earthquake-prone regions.

This study has several limitations that should be acknowledged. Additional plots such as strain distributions, stress redistributions, and crack maps were not included due to space considerations, although they could provide further insight into the underlying mechanisms. Similarly, residual drift, cumulative energy dissipation, and damage indices were not assessed but would enrich the evaluation of seismic performance. Future research should therefore integrate these parameters together with more refined bond–slip models to better capture local debonding and pinching effects and consider cyclic deterioration of CFRP and steel materials to more realistically simulate energy dissipation under repeated loading. Moreover, large-scale studies are needed to validate the applicability of NSM-CFRP retrofitting, while investigations on long-term performance under environmental exposure and fatigue are essential to ensure durability and reliability.

## Figures and Tables

**Table 1:** Description of beam specimens considered in the study.

Specimen ID	Prestressing Level	CFRP Strengthening Configuration
BN-L	35% (Low)	-
BN-H	70% (High)	
BH-L	35% (Low)	Half-embedded CFRP rods
BH-H	70% (High)	
BF-L	35% (Low)	Fully embedded CFRP rods
BF-H	70% (High)	

**Table 2:** The CDP parameter of plasticity.

Dilation Angle (ψ)	Eccentricity (e)	$\sigma_{b0}/\sigma_{c0}$	$K_{c}$	Viscosity Parameter
35°	0.1	1.16	2/3	0.0005

**Table 3:** Material properties of CFRP sheets.

Properties	Value
Tensile strength along fiber direction, $X_{1}^{T}$ (MPa)	3850
Compressive strength along fiber direction, $X_{1}^{C}$ (MPa)	−2369.23
Young’s modulus along fiber direction, $E_{11}$ (GPa)	225
Tensile strength perpendicular to fiber direction, $X_{2}^{T}$ (MPa)	109.58
Compressive strength perpendicular to fiber direction, $X_{2}^{C}$ (MPa)	−286.28
Young’s modulus perpendicular to fiber direction, $E_{22}$ (GPa)	16.28
Shear strength, $S_{12}$ (MPa)	118.46
Shear modulus, $G_{12}$ (GPa)	4.31

**Table 4:** Cohesive stiffness parameters adopted for the half-embedded CFRP rod model.

Parameter	Description	Value (N/mm^3^)
*K_nn_*	Normal penalty stiffness	3000
*K_ss_*	Shear penalty stiffness in first direction	8
*K_tt_*	Shear penalty stiffness in second direction	8

**Table 5:** Comparison between experimental and numerical results.

Specimen	Experimental [41]		Numerical		% Difference	
	*Pu* (kN)	Δ*_u_* (mm)	*Pu* (kN)	Δ*_u_* (mm)	*Pu*	Δ*_u_*
BN	158.36	25.90	150.78	25.30	4.79	2.35
BH	197.06	14.49	183.09	14.46	7.09	0.17
BF	217.88	25.81	214.93	25.29	1.36	2.03

**Table 6:** Displacement ductility of analyzed specimens.

Specimen	Positive Direction		Negative Direction		Ductility (*µ*)
	$\Delta_{y}^{+}$ (mm)	$\Delta_{u}^{+}$ (mm)	$\Delta_{y}^{-}$ (mm)	$\Delta_{u}^{-}$ (mm)	
**Low level of initial prestressing**					
BN-L-C	4.14	18.25	−7.37	−18.20	6.90
BH-L-C	4.06	18.25	−6.87	−18.20	7.10
BF-L-C	4.09	18.25	−7.13	−18.20	7.10
**High level of initial prestressing**					
BN-H-C	3.73	18.25	−7.82	−18.20	7.20
BH-H-C	3.65	18.25	−7.89	−18.20	7.30
BF-H-C	3.72	18.25	−8.26	−18.20	7.10

## Data Availability

The original contributions presented in this study are included in the article. Further inquiries can be directed to the corresponding authors.

## Abbreviation

The following abbreviations are used in this manuscript:

RC	Reinforced concrete
FRP	Fiber-reinforced polymer
EB	Externally bonded
NSM	Near-surface mounted
CFRP	Carbon fiber-reinforced polymer
FE	Finite element
FEM	Finite element method
SRC	Steel-reinforced concrete
ECC	Engineered cementitious composites
CDP	Concrete damage plasticity
NMSE	Normalized mean square error
